# Genome-wide insights into genetic diversity of endemic and non-endemic *Ixodes ricinus* populations

**DOI:** 10.1038/s41598-025-15640-2

**Published:** 2025-10-01

**Authors:** Olcay Hekimoğlu, İsmail Kudret Sağlam

**Affiliations:** 1 Faculty of Science, Department of Biology, Division of Ecology, Hacettepe University, 06800 Beytepe, Ankara, Turkey; 2 Faculty of Science, Department of Molecular Biology and Genetics, Koç University, 34450 İstanbul, Turkey

**Keywords:** *Ixodes ricinus*, Türkiye, Whole genome sequencing, Genomic diversity, Comparative genomics, Genome evolution, Evolutionary genetics, Population genetics

## Abstract

*Ixodes ricinus*, a major vector of tick-borne pathogens in Europe, exhibits considerable geographic variation in genetic structure and vector competence. This study investigates genome-wide polymorphism and population structure in *I. ricinus* populations from Türkiye -where tick-borne encephalitis (TBE) is not endemic—compared to populations from Germany and Algeria, for which genomic data are publicly available. Whole-genome sequencing was performed on 15 individuals from four geographically distinct Turkish populations and evaluated with German and Algerian samples available in GenBank. Principal component analysis (PCA) and ADMIXTURE clustering revealed clear genetic separation between Turkish and German populations. Genomic diversity scans identified 37 highly differentiated genomic regions, several of which contained genes associated with detoxification, lipid metabolism, chemosensory processes, and ion transport—functions relevant to vectorial capacity and local adaptation. Gene Ontology enrichment analyses supported these findings, highlighting overrepresentation of carotene and terpene metabolism pathways. Notably, Turkish populations exhibited more admixture and heterogeneity than the genetically homogeneous German population. These results highlight region-specific genomic differentiation in *I. ricinus*, which may influence ecological interactions and vector-associated traits.

## Introduction

*Ixodes ricinus*, commonly known as the sheep tick, is a significant member of the *Ixodes ricinus* complex, which consists of 14 species found worldwide^1,2^. It is particularly prevalent in Europe and is recognized as the most widely distributed tick species on the continent^3^. This tick species is a generalist that feeds on a wide range of wildlife hosts^4,5^. *I. ricinus* is the vector of several diseases, including Lyme borreliosis, anaplasmosis and tick-borne encephalitis (TBE)^6,7^.

*Ixodes ricinus* is the most extensively studied tick species in Europe^3^. Data from these studies have revealed the existence of genetically distinct populations of the species^8–12^. Earlier studies based on microsatellite loci also detected population differentiation in *I. ricinus* in the Baltic region^13^. In particular, a distinct genetic pattern was observed in North African populations^10^, leading to the reclassification of this taxon as *Ixodes inopinatus*^14^. These studies indicate that there is still confusion and uncertainty regarding the number of genetic groups of *I. ricinus* worldwide and their species status. This highlights the need for more detailed analyses based on genomic data^15^. In support of this view, genomic data-based comparisons between *I. inopinatus* and *I. ricinus* in Germany revealed that individuals previously identified as *I. inopinatus* were in fact *I. ricinus*^16^. Furthermore, another study conducted in TBE-endemic and non-endemic areas demonstrated that geographical and genetic differences in ticks may influence their susceptibility to specific virus strains^17,18^. Therefore, advanced genomic analyses are needed to gain a more comprehensive understanding of the genetic diversity and vectorial capacity of *I. ricinus*.

High-quality genomic data enable the detection of cryptic species, shifts in vector distributions, and adaptive evolutionary changes, all of which are integral to a comprehensive understanding of disease transmission dynamics^19,20^. The sequencing and assembly of the first tick genome, *Ixodes scapularis* enabled studies on the mechanisms underlying tick‒host‒pathogen interactions, thereby clarifying many unknown aspects of tick biology^21^. Additionally, using this reference genome, the entire genome of *I. ricinus* was sequenced and assembled^22,23^. Following this, the "1000 *Ixodes* Genome Project" (Ix100G) was announced, aiming to expand the genetic resources of *Ixodes* species and collect data from different populations^15^. In addition, genome assembly and annotation of *I. scapularis* have been produced using diverse technologies^24,25^. More recently, a study analyzing the genome sequences of four *Ixodes* species provided significant insights into tick evolution and genomic features, with the *I. ricinus* genome assembled at the chromosomal level. Notable expansions were observed in gene families, particularly in regions associated with detoxification, lipid metabolism, and chemosensory mechanisms^26^. This study highlighted the specialization of different genomic regions for specific functions^26^. It has also been demonstrated that transposable elements constitute the majority of the *I. ricinus* genome^26,27^, which could facilitate adaptation to different environmental conditions and various host species^26^. Recent whole-genome and deep sequencing studies have provided novel insights into *I. ricinus* and *I. inopinatus*, highlighting their genomic similarity^28^, the structure and transmission dynamics of endosymbionts^29^, and geographical variation in tick-associated microbiota that may influence vector competence^30^. These findings underscore the broader utility of genome-wide approaches for understanding vector biology and host–microbe interactions.

The extent to which genetic differences among *I. ricinus* populations influence their role as disease vectors, the impact of these differences on pathogen transmission, and the mechanisms driving disease emergence remain critical but unresolved questions. Although *Ixodes*-borne diseases are widespread in Europe and the presence and distribution of *I. ricinus* has been documented in northern parts of Türkiye^31–34^, the relatively low number of Lyme disease cases and the absence of tick-borne encephalitis virus (TBEV) in Türkiye are quite remarkable. This study investigates genome-wide polymorphism and diversity patterns in *I. ricinus* populations from Türkiye and Germany to identify genomic regions exhibiting significant divergence in nucleotide diversity. By integrating functional annotation and gene ontology analysis, we aim to determine whether these divergent regions are associated with genes involved in pathways relevant to vector competence, metabolism, and ecological adaptation. To achieve this, we analyzed whole-genome data from *I. ricinus* individuals sampled across different populations in Türkiye and compared them to publicly available *I. ricinus* sequences from Germany and *I. inopinatus* samples from Algeria in GenBank for comparative context. German populations were selected because they represent a well-characterized, genetically homogeneous European group from a TBE-endemic region, allowing comparison with non-endemic Turkish populations. The Algerian populations were included to provide additional evolutionary context, as they represent North African lineages where the taxonomic distinction between *I. ricinus* and *I. inopinatus* remains under debate. Furthermore, the vectorial competence of *I. inopinatus* for TBE and Lyme disease has not been established, which allows for a broader comparison of potential differences in vectorial capacity and ecological adaptation.

## Materials and methods

### Sample selection and DNA extraction

**Fig. 1:**
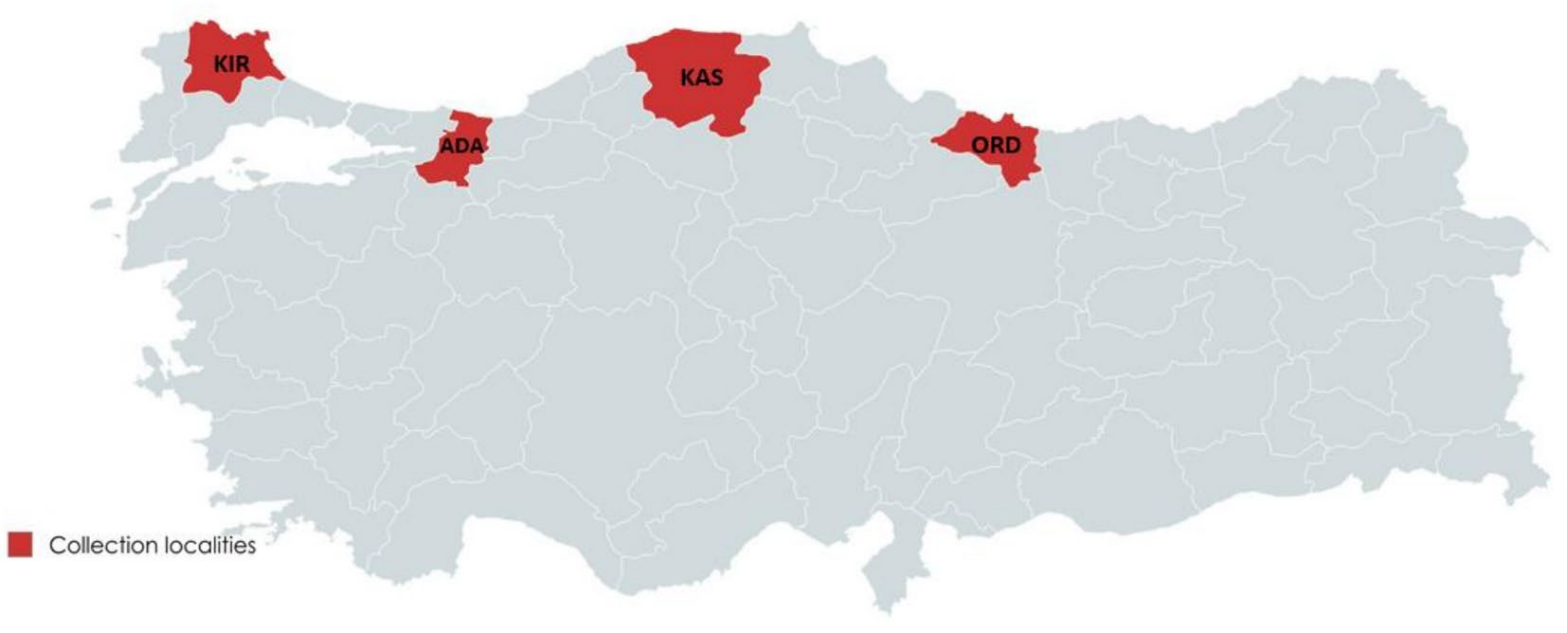
Sampling sites of *Ixodes ricinus* populations across northern Türkiye.

The selection of individuals for DNA extraction was designed to represent the spatial distribution of *I. ricinus* across Türkiye, considering both the number of specimens collected per site and the quality of extracted DNA. Accordingly, DNA isolation was performed on a total of 114 adult individuals. A subset of 15 samples was selected for whole-genome sequencing based on two criteria: (i) high DNA concentration and integrity as measured by Qubit fluorometry, and (ii) balanced representation of the four geographic populations. Samples with low DNA quality or degraded material were excluded from further genomic analysis. Ultimately, whole-genome data were obtained from 15 individuals representing four populations, including five from Kırklareli (Northwest/Thrace) population, four from Adapazarı (Marmara) population, three from Kastamonu (Western Black Sea) population, and three from Ordu (Central Black Sea) population (Fig. 1; Supplementary Table 1). Morphological identification was conducted using taxonomic keys^35,36^. Prior to DNA extraction, all tick specimens were preserved in 70% ethanol and stored at − 20 °C.

Genomic DNA was extracted from the entire tick body using the DNeasy Blood & Tissue Kit (QIAGEN) and the GeneJET Genomic DNA Purification Kit (Thermo Fisher Scientific), which have been reported to yield the highest DNA concentrations from *Ixodes* ticks^37^. RNase A (Thermo Fisher Scientific) was added during the extraction process to remove residual RNA. The quality of the extracted DNA was assessed using a Qubit 3 Fluorometer (Thermo Fisher Scientific) and the Qubit dsDNA HS Assay Kit (Thermo Fisher Scientific). Some *I. ricinus* individuals in this study were collected from hosts and were semi-engorged. To minimize the risk of host DNA contamination, all genomic reads were aligned to the *I. ricinus* reference genome, and non-matching reads were discarded during the bioinformatics processing steps. This strategy ensures that only tick-derived sequences were retained for variant calling and downstream analyses. High-quality DNA samples were subsequently used for whole-genome library preparation with an average insert size of 350 bp, performed at Novogene Europe (Cambridge, UK). Paired-end sequencing (2 × 150 bp) was conducted on the Illumina NovaSeq 6000 platform, targeting a minimum of 10X coverage per sample.

### Alignment to the Reference Genome

Raw sequencing data quality was assessed using the FastQC v0.11.9 (https://github.com/s-andrews/FastQC). For alignment, the reference genome of *I. ricinus* was downloaded from BioInformatics Platform for Agroecosystem Arthropods (https://bipaa.genouest.org/is/ticks/) using Annotation OGS1.3^26^. This genome corresponds to the chromosomal-level assembly published by Cerqueira de Araujo et al. (2025) and is available under GenBank BioProject accession number PRJEB67792. A total of 23 samples—15 from Türkiye, six from Germany (GenBank Accs. numbers: SRR23586399—SRR23586404), two from Algeria (GenBank Accs. numbers: SRR23586405—SRR23586406)^16^—were aligned to the reference genome. Alignment was performed using the Burrows-Wheeler Alignment (BWA) tool with the BWA-MEM algorithm, which ensures accurate and efficient mapping^38^. Aligned reads were converted to BAM format using SAMTOOLS v1.14^39^. Further processing steps, including sorting, filtering by proper pairs, marking of PCR duplicates, and indexing, were performed using the same program. Alignment statistics relative to the reference genome were computed using the “samtools flagstat” function. Alignment metrics for all samples are presented in Supplementary Table 2.

### Genotype likelihood estimation and high-quality site selection

We used Analysis of Next Generation Sequencing Data (ANGSD)^40^ to identify high-confidence variant sites for downstream analysis. Genotype likelihoods were calculated under the SAMtools model (-GL 1), inferred major and minor (-doMajorMinor 1) and estimated minor allele frequencies (-doMaf 1). Stringent quality filters were applied, retaining only sites with a minimum base quality of 30 (-minQ 30), mapping quality of at least 10 (-minMapQ 10), presence in at least half of the individuals in each population (-minInd), and an average per individual read depth of 10X (-setMinDepth). Following site filtering, we conducted genotype calling and single nucleotide polymorphism (SNP) detection using ANGSD^40^. Genotype likelihoods were output in binary format (-doGlf 2), and posterior genotype probabilities were computed (-doPost 2) with a posterior cutoff threshold of 0.95 (-postCutoff 0.95). Genotype calls were obtained (-doGeno 5), and results were exported into both BCF (-doBcf 1) and PLINK (-doPlink 2) formats. In addition, we applied a minimum allele frequency filter of 0.05 (-minMaf 0.05) and a stringent SNP significance threshold (-SNP_pval 1e-12) to ensure a robust and high-confidence variant dataset.

### Genetic structure and ancestry inference

Principal component analysis (PCA) was performed to investigate the genetic structure among populations. Eigenvalues and eigenvectors were computed using PCAone^41^ with genotype likelihood data in Beagle format, specifying the extraction of the top 10 principal components (-k 10). The PCA was conducted to explore genetic structure among 20 *I. ricinus* individuals from four Turkish populations—Kırklareli (KIR), Adapazarı (ADA), Kastamonu (KAS), and Ordu (ORD)—along with German (GER) and Algerian (ALG) populations^16^. Principal component axes summarizing genetic variation were derived from the covariance matrix using eigenvalue decomposition in R v4.2.0^42^, and the first two components were subsequently plotted to visualize population structure using ggplot2^43^.

Admixture proportions and individual ancestries were estimated using NGSadmix^44^, which infers population structure based on genotype likelihoods. Analyses were performed for a range of hypothetical ancestral populations (K = 2 to K = 4), with ten independent runs per K value to assess convergence and consistency. Each run was initialized with a different random seed. Input data consisted of genotype likelihoods in beagle format, and analyses were executed using four computational threads per run. The results, including estimated admixture proportions and model likelihoods, were stored for downstream visualization and model comparison.

### Runs of homozygosity (ROH) analysis

Whole-genome BAM files were processed to generate variant call format (VCF) files using samtools v1.14 and *bcftools* v1.14^39^. Variant calling was performed with bcftools mpileup followed by bcftools call, and the resulting VCF files were normalized using bcftools norm to ensure consistency with the reference genome. Normalized VCF files were converted into PLINK binary format (.bed/.bim/.fam) using PLINK v1.90^45^ with options to allow scaffolds as chromosomes.

ROH analyses were conducted in PLINK using relaxed thresholds to capture shorter and low-SNP ROH segments: a minimum of 50 SNPs, a minimum ROH length of 500 kb, no heterozygous sites allowed within a sliding window of 50 SNPs, and a maximum gap of 500 kb between SNPs within a ROH. Separate analyses were performed for each population.

### Genetic diversity

To assess genetic diversity differences between *I. ricinus* populations from Türkiye and Germany, we analyzed genome-wide polymorphism patterns using whole-genome sequencing data. Genetic diversity was estimated by calculating pairwise nucleotide differences (Theta Pi, π) in 50 kb genomic windows with a step size of 25 kb across all chromosomes and standardized these values to per-site estimates. Theta Pi values were estimated in ANGSD based on the site frequency spectrum (SFS) using *I. inopinatus* as the outgroup by first calculating allele frequency likelihoods (-doSaf 1) followed by maximum likelihood estimation of the SFS using the realSFS module^46^.

To identify genomic regions where Turkish populations exhibited the highest differences in diversity compared to German populations, we first extracted diversity estimates for *I. ricinus* individuals from Germany and used these as a reference. We then computed the difference in Theta Pi between each Turkish population and the German reference for each genomic window. These calculations were performed separately for each chromosome. Regions exhibiting significant deviations in diversity were identified based on a threshold of two standard deviations from the mean difference in Theta Pi. To assess the statistical significance of these deviations, we performed one-sample t-tests comparing the observed differences to a mean of zero, retaining only regions with at least two observations.

The identified regions were recorded and visualized for each chromosome, with diversity differences plotted along genomic coordinates. All statistical analyses and visualizations were conducted using R (version 4.2.0) with the ggplot2^43^, dplyr (https://dplyr.tidyverse.org), and tidyr (https://tidyr.tidyverse.org) packages.

### Gene ontology (GO) analysis

For each genomic region exhibiting high differentiation between *I. ricinus* populations from Germany and Türkiye, we identified the nearest gene accessions within a 100 kb window based on the *I. ricinus* reference genome. Protein sequences of these genes were then cross-referenced against *I. scapularis* annotations in VectorBase (https://vectorbase.org/) using BLAST to identify homologous orthologs for downstream gene ontology (GO) analysis.

GO enrichment analysis was conducted in R using the clusterProfiler v4.0^47^, with the *I. scapularis* annotation database serving as the background. Biological Process (BP) terms were tested for overrepresentation using a hypergeometric test, with multiple testing correction applied via the Benjamini–Hochberg method. GO terms with a false discovery rate (FDR) < 0.2 and a raw p-value < 0.05 were considered significantly enriched.

## Results

### Mapping and alignment statistics

A total of 15 individual libraries from four distinct populations in Türkiye (Adapazarı [ADA], Kastamonu [KAS], Kırklareli [KIR], and Ordu [ORD]), as well as samples from Algeria (ALG) and Germany (GER), were investigated. Raw read counts ranged between 62 million to 1.23 billion per sample. Turkish samples showed consistently high mapping rates, with 90–98% of reads aligning properly to the reference genome. The highest alignment efficiencies were observed in individuals from the KIR and ORD populations (up to 97.7%). German samples exhibited more variable mapping success, ranging from 57.4 to 82.7% (Supplementary Table 2).

### PCA and admixture

**Fig. 2:**
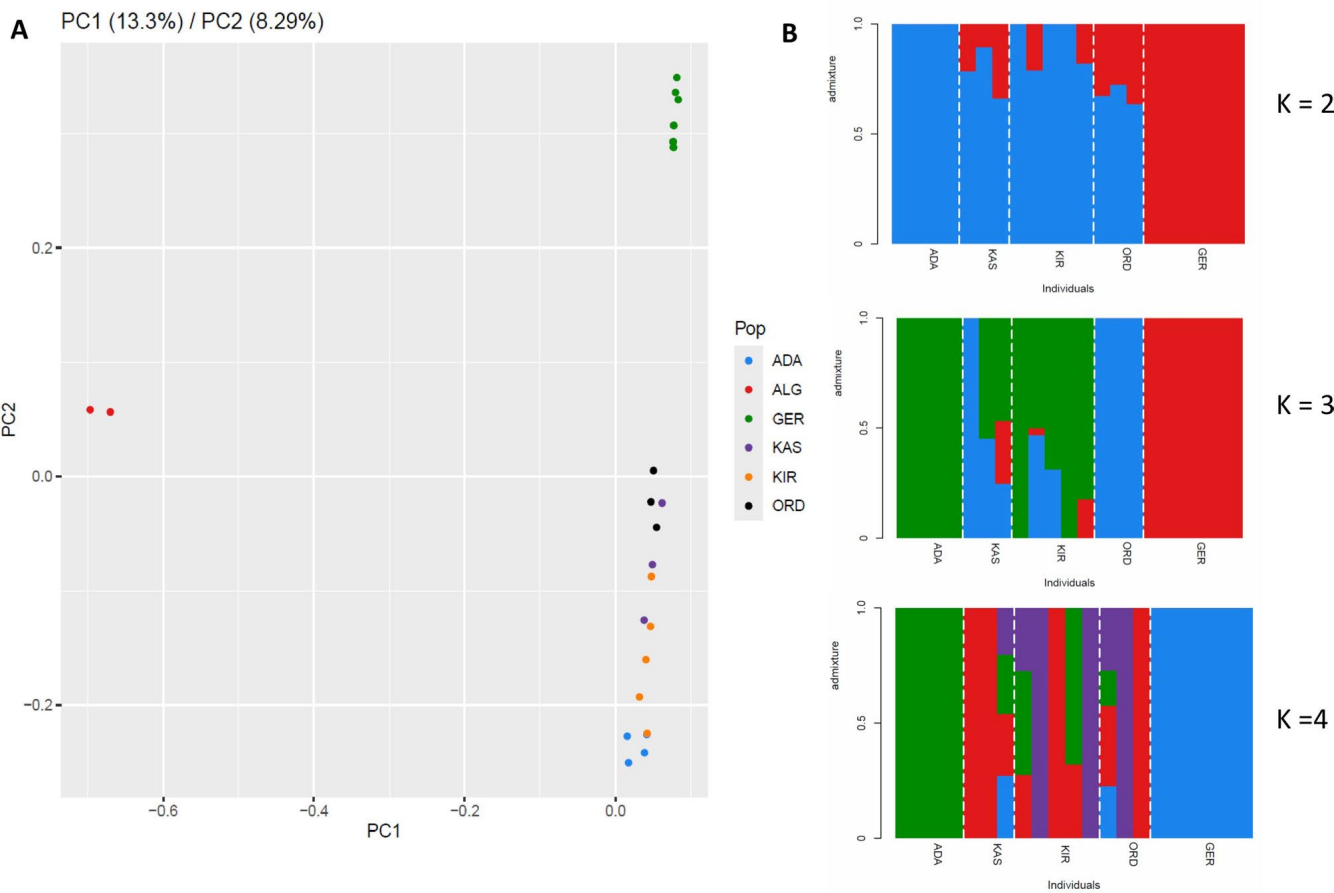
Genetic structure of *I. ricinus* populations. (**A**) Principal component analysis (PCA) illustrating genetic differentiation among populations based on genome-wide SNP data. (**B**) ADMIXTURE analysis displaying individual ancestry proportions for K = 2, 3, and 4, highlighting patterns of genetic clustering across populations.

**PCA.** The first two principal components explained 13.3% (PC1) and 6.29% (PC2) of the total genetic variation, respectively (Fig. 2A). PC1 primarily separates the Algerian population (ALG) from both the Turkish and German samples, consistent with its taxonomic distinction as *I. inopinatus*. Due to this clear interspecific separation, the Algerian samples (*I. inopinatus*) were not included in the genetic diversity (Theta Pi) analyses, which focused specifically on within-species comparisons between Turkish and German *I. ricinus* populations. PC2, on the other hand, clearly differentiates the Turkish populations (ADA, KAS, KIR, ORD) from the German population, reflecting substantial genetic divergence between these groups. This suggests a degree of genetic differentiation between German and the Turkish samples, potentially reflecting geographic isolation or limited gene flow (Fig. 2A). Additionally, genetic separation among Turkish populations along PC2 suggests the presence of geographic structuring within Türkiye.

**Admixture** analysis revealed that the model assuming three ancestral populations (K = 3) best explains the genetic structure of the samples. Individuals exhibited varying levels of ancestry proportions assigned to the three inferred genetic clusters. The GER population displayed a high degree of genetic homogeneity, with most individuals predominantly assigned to a single ancestry. In contrast, individuals from the Turkish populations (ADA, KAS, KIR, ORD) exhibited more heterogeneous ancestry profiles, with varying degrees of contribution from two or more clusters. Among the Turkish populations, KAS and KIR exhibited relatively high levels of admixture,whereas ADA showed mono-ancestry at both K=3 and K=4. ORD was also mono-ancestral at K=3 but displayed appreciable admixture at K=4.

The overall pattern is consistent with the separation observed in the PCA, where German individuals formed a distinct cluster relative to Turkish samples.

### ROH of Turkish populations

Across all Turkish *I. ricinus* populations analyzed, no ROH segments exceeding 500 kb were identified, indicating a lack of extended homozygous regions. This pattern was consistent across the four regional populations (KIR, ADA, KAS, and ORD), suggesting overall high levels of heterozygosity.

### Genetic diversity

**Fig. 3:**
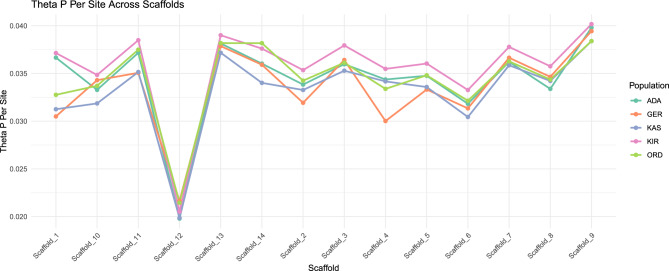
Mean genome-wide nucleotide diversity (Theta Pi) across all 14 major scaffolds for Turkish and German *I. ricinus* populations.

**Fig. 4:**
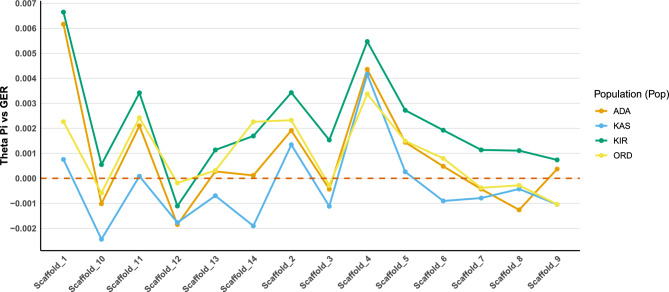
Mean differences in nucleotide diversity (Theta Pi) between Turkish and German *I. ricinus* populations across the 14 major scaffolds.

Genome-wide mean nucleotide diversity (Theta Pi) values across all 14 major scaffolds were generally similar among populations, typically ranging from 0.03 to 0.04 per site (Table 1, Fig. 3). The only notable exception was Scaffold 12, which consistently exhibited lower diversity across all populations (0.018–0.020 per site). Overall, mean differences in nucleotide diversity between Turkish and German populations were relatively small (~ 0.004) yet statistically significant (Supplementary Table 3). Furthermore, nucleotide diversity consistently tended to be higher in Turkish populations compared to the German population (Fig. 4). The most pronounced divergence in mean Theta Pi was observed on Scaffold 4, where Turkish populations displayed substantially higher nucleotide diversity compared to the German reference (Fig. 4).

**Table 1:** Genome-wide nucleotide diversity (Theta Pi) and Tajima’s D values for Turkish and German populations of *Ixodes ricinus* across all 14 major scaffolds.

Chr	Pop	Theta Pi per site		Tajima’s D	
		Mean	Stdev	Mean	Stdev
Scaffold_1	ADA	0.0326	0.0102	-0.3318	0.3158
	GER	0.0235	0.0082	-0.8066	0.2209
	KAS	0.0292	0.0092	-0.2491	0.2258
	KIR	0.0298	0.0091	-0.7438	0.2441
	ORD	0.0302	0.0098	-0.2858	0.1933
Scaffold_2	ADA	0.0310	0.0083	-0.3911	0.2081
	GER	0.0272	0.0087	-0.5809	0.2404
	KAS	0.0324	0.0083	-0.2232	0.1848
	KIR	0.0309	0.0083	-0.5417	0.2276
	ORD	0.0334	0.0088	-0.2343	0.1787
Scaffold_3	ADA	0.0307	0.0135	-0.5085	0.2462
	GER	0.0271	0.0125	-0.8258	0.2786
	KAS	0.0322	0.0137	-0.2773	0.2407
	KIR	0.0302	0.0131	-0.7121	0.2511
	ORD	0.0324	0.0134	-0.3234	0.2390
Scaffold_4	ADA	0.0307	0.0093	-0.3767	0.2324
	GER	0.0240	0.0084	-0.7197	0.2345
	KAS	0.0325	0.0094	-0.2076	0.1840
	KIR	0.0302	0.0085	-0.5954	0.2218
	ORD	0.0319	0.0087	-0.2733	0.1750
Scaffold_5	ADA	0.0310	0.0081	-0.4534	0.2509
	GER	0.0263	0.0075	-0.7841	0.2512
	KAS	0.0317	0.0084	-0.2943	0.2118
	KIR	0.0306	0.0082	-0.5982	0.2728
	ORD	0.0329	0.0084	-0.3065	0.2160
Scaffold_6	ADA	0.0288	0.0079	-0.4489	0.2184
	GER	0.0255	0.0073	-0.7420	0.1797
	KAS	0.0299	0.0082	-0.1817	0.2254
	KIR	0.0289	0.0081	-0.5489	0.2446
	ORD	0.0311	0.0079	-0.2560	0.1560
Scaffold_7	ADA	0.0317	0.0114	-0.4945	0.1840
	GER	0.0288	0.0113	-0.7318	0.1954
	KAS	0.0338	0.0121	-0.2674	0.1466
	KIR	0.0311	0.0115	-0.6675	0.1748
	ORD	0.0344	0.0115	-0.2568	0.1430
Scaffold_8	ADA	0.0299	0.0076	-0.4888	0.2101
	GER	0.0287	0.0072	-0.6313	0.2933
	KAS	0.0324	0.0074	-0.3312	0.1846
	KIR	0.0300	0.0077	-0.6930	0.2390
	ORD	0.0326	0.0081	-0.2953	0.1999
Scaffold_9	ADA	0.0325	0.0115	-0.4889	0.1891
	GER	0.0289	0.0107	-0.7848	0.2028
	KAS	0.0341	0.0121	-0.2558	0.1842
	KIR	0.0318	0.0116	-0.6572	0.2165
	ORD	0.0338	0.0119	-0.2812	0.1811
Scaffold_10	ADA	0.0290	0.0070	-0.5610	0.1736
	GER	0.0263	0.0067	-0.8658	0.1816
	KAS	0.0301	0.0071	-0.3175	0.1733
	KIR	0.0287	0.0069	-0.7410	0.1686
	ORD	0.0316	0.0071	-0.3413	0.1743
Scaffold_11	ADA	0.0347	0.0106	-0.4637	0.2176
	GER	0.0286	0.0092	-0.7903	0.1918
	KAS	0.0341	0.0099	-0.3069	0.2047
	KIR	0.0339	0.0102	-0.6334	0.2315
	ORD	0.0363	0.0104	-0.3881	0.1769
Scaffold_12	ADA	0.0183	0.0041	-0.4484	0.1179
	GER	0.0183	0.0038	-0.6575	0.1222
	KAS	0.0196	0.0042	-0.2568	0.1145
	KIR	0.0180	0.0038	-0.6217	0.1304
	ORD	0.0209	0.0042	-0.2887	0.1192
Scaffold_13	ADA	0.0342	0.0129	-0.3869	0.2033
	GER	0.0308	0.0113	-0.6553	0.2156
	KAS	0.0357	0.0130	-0.1617	0.2068
	KIR	0.0336	0.0126	-0.5160	0.2451
	ORD	0.0364	0.0118	-0.1890	0.1857
Scaffold_14	ADA	0.0319	0.0108	-0.4079	0.1728
	GER	0.0284	0.0100	-0.6838	0.2127
	KAS	0.0327	0.0103	-0.1302	0.2139
	KIR	0.0320	0.0103	-0.5469	0.1993
	ORD	0.0361	0.0114	-0.2436	0.1733

To identify the genomic regions contributing most to the observed differentiation in diversity, we conducted one-sample *t*-tests for each genomic window, comparing the difference in Theta Pi between Turkish and German populations to a null expectation of zero (P < 10⁻⁶). We retained regions that were significant in at least three out of four Turkish populations and consistent across scaffolds (Supplementary Tables 4–7). This analysis revealed 61 non-overlapping outlier windows distributed across the 14 major scaffolds of the *I. ricinus* genome. These windows ranged from 25 to 50 kb in size and were particularly enriched on Scaffolds 1, 4, 6, and 13. Several of these outlier regions occurred as consecutive windows within larger genomic intervals. For example, between ~ 180.05 and 180.20 Mb on Scaffold 1, and between ~ 86.27 and 86.45 Mb on Scaffold 13. This suggests the presence of extended haplotypes or genomic blocks characterized by coordinated shifts in nucleotide diversity.

### Genomic regions of divergence and functional enrichment (GO Analysis)

A total of 61 genomic windows showing high differentiation between Turkish and German *I. ricinus* populations were linked to 37 nearby genes within a 50 kb window (Table 2). Homologs of these genes were identified in the *I. scapularis* genome and used for downstream ontology analysis. Several candidate genes were located within these highly divergent intervals. On Scaffold 1, a cluster of adjacent genes—IricT00000373 to IricT00000376—was annotated as encoding carotenoid oxygenases (InterPro: IPR004294), enzymes potentially involved in oxidative metabolism and detoxification. These genes share orthology with *I. scapularis* sequences such as XP_002412141 and EEC14724, indicating conservation across species. Another gene of interest, IricT00001060, was associated with GO terms related to cell morphogenesis and developmental regulation, with its *I. scapularis* ortholog (XP_040071071) supporting possible roles in structural or signaling functions. In addition, the adjacent genes IricT00001480 and IricT00001481, both encoding neurotransmitter-gated ion channels, were located in divergent genomic regions and annotated with GO terms related to ion transport and transmembrane signaling. Their differentiation may reflect population-level variation in sensory processing or host-seeking behavior, potentially contributing to ecological adaptation.

**Table 2:** Genomic windows showing high differentiation between Turkish and German *I. ricinus* populations and their associated genes. For each window, scaffold ID, start and end positions, nearby *I. ricinus* genes (within or up to 50 kb), and corresponding homologs in the *I. scapularis* (Wikel) reference genome are listed.

Chromosome	Start	End	Genes	Homologs in *I. scapularis*, Wikel
Iricinus_Scaffold_1	40,250,000	40,300,000	IricT00000374—within the interval; IricT00000373—15278 bp upstream;IricT00000375—29878 bp downstream; IricT00000376—33345 bp downstream	ISCW021870; ISCW021869; ISCW021870; ISCW021869
Iricinus_Scaffold_1	114,100,000	114,150,000	IricT00001060—within the interval	ISCW003216
Iricinus_Scaffold_1	114,125,000	114,175,000	IricT00001061—within the interval; IricT00001062—41499 bp downstream	ISCW013096
Iricinus_Scaffold_1	157,975,000	158,025,000	IricT00001481—within the interval; IricT00001482—37186 bp downstream	ISCW003226; ISCW011253
Iricinus_Scaffold_1	158,000,000	158,050,000	IricT00001480—23795 bp upstream; ricT00001482—12186 bp downstream	ISCW014321
Iricinus_Scaffold_1	170,050,000	170,100,000	IricT00001595—36943 bp downstream	ISCW018541
Iricinus_Scaffold_1	170,075,000	170,125,000	IricT00001595—11943 bp downstream	ISCW018541
Iricinus_Scaffold_1	180,025,000	180,075,000	IricT00001657—34773 bp upstream	ISCW007904
Iricinus_Scaffold_1	180,050,000	180,100,000	NA	NA
Iricinus_Scaffold_1	180,100,000	180,150,000	NA	NA
Iricinus_Scaffold_1	180,125,000	180,175,000	IricT00001658—8491 bp upstream; IricT00001659—35749 bp downstream	ISCW010683
Iricinus_Scaffold_1	180,150,000	180,200,000	IricT00001658—33491 bp upstream; IricT00001659—10749 bp downstream	ISCW010683
Iricinus_Scaffold_1	180,250,000	180,300,000	NA	NA
Iricinus_Scaffold_1	180,275,000	180,325,000	IricT00001659—23903 bp upstream	ISCW010683
Iricinus_Scaffold_2	94,825,000	94,875,000	IricT00010185—23878 bp downstream; IricT00010186—41794 bp downstream	ISCW017666; ISCW017663
Iricinus_Scaffold_3	92,750,000	92,800,000	IricT00011392—within the interval; IricT00011393—43972 bp downstream	ISCW006655; ISCW024732
Iricinus_Scaffold_3	92,775,000	92,825,000	IricT00011393—18972 bp downstream	ISCW024732
Iricinus_Scaffold_3	140,325,000	140,375,000	IricT00011610—36162 bp downstream	NA
Iricinus_Scaffold_3	140,825,000	140,875,000	NA	NA
Iricinus_Scaffold_4	37,450,000	37,500,000	NA	NA
Iricinus_Scaffold_4	54,100,000	54,150,000	IricT00012793—35496 bp upstream; IricT00012794—30805 bp upstream; IricT00012795—30803 bp upstream	ISCW012733; ISCW024918; ISCW024143
Iricinus_Scaffold_4	111,375,000	111,425,000	NA	NA
Iricinus_Scaffold_4	165,675,000	165,725,000	NA	NA
Iricinus_Scaffold_4	165,700,000	165,750,000	IricT00014105—16866 bp upstream	ISCW000275
Iricinus_Scaffold_4	165,725,000	165,775,000	IricT00014105—41866 bp upstream	ISCW000275
Iricinus_Scaffold_4	165,750,000	165,800,000	NA	NA
Iricinus_Scaffold_4	165,775,000	165,825,000	NA	NA
Iricinus_Scaffold_5	60,100,000	60,150,000	IricT00015246—20051 bp downstream; IricT00015247—39880 bp downstream	ISCW007041; ISCW009048
Iricinus_Scaffold_5	86,150,000	86,200,000	NA	NA
Iricinus_Scaffold_5	120,600,000	120,650,000	IricT00015627—46210 bp downstream	ISCW014313
Iricinus_Scaffold_5	120,625,000	120,675,000	IricT00015627—21210 bp downstream	ISCW014313
Iricinus_Scaffold_6	96,000,000	96,050,000	IricT00016898—42925 bp upstream	ISCW019198
Iricinus_Scaffold_6	96,025,000	96,075,000	IricT00016900—29946 bp downstream	ISCW014963
Iricinus_Scaffold_6	159,300,000	159,350,000	NA	NA
Iricinus_Scaffold_6	159,675,000	159,725,000	IricT00017774—4564 bp upstream	ISCW000431
Iricinus_Scaffold_6	159,700,000	159,750,000	IricT00017774—29564 bp upstream	ISCW000431
Iricinus_Scaffold_6	159,825,000	159,875,000	NA	NA
Iricinus_Scaffold_6	159,900,000	159,950,000	NA	NA
Iricinus_Scaffold_6	159,925,000	159,975,000	NA	NA
Iricinus_Scaffold_7	106,050,000	106,100,000	NA	NA
Iricinus_Scaffold_7	106,075,000	106,125,000	NA	NA
Iricinus_Scaffold_7	106,100,000	106,150,000	IricT00019101—34668 bp downstream	ISCW001390
Iricinus_Scaffold_7	106,125,000	106,175,000	IricT00019101—9668 bp downstream	ISCW001390
Iricinus_Scaffold_7	106,150,000	106,200,000	NA	NA
Iricinus_Scaffold_7	113,500,000	113,550,000	IricT00019253—8588 bp upstream	ISCW013261
Iricinus_Scaffold_8	44,525,000	44,575,000	NA	NA
Iricinus_Scaffold_10	50,175,000	50,225,000	IricT00003002—8368 bp upstream	ISCW011215
Iricinus_Scaffold_10	101,000,000	101,050,000	NA	NA
Iricinus_Scaffold_11	56,550,000	56,600,000	NA	NA
Iricinus_Scaffold_12	15,900,000	15,950,000	NA	NA
Iricinus_Scaffold_12	24,125,000	24,175,000	NA	NA
Iricinus_Scaffold_12	52,250,000	52,300,000	IricT00005264—30140 bp downstream	ISCW000942
Iricinus_Scaffold_13	78,300,000	78,350,000	IricT00007253—46501 bp upstream; IricT00007255—4984 bp downstream; IricT00007256—6122 bp downstream	ISCW003282; ISCW012472; ISCW002757
Iricinus_Scaffold_13	84,950,000	85,000,000	IricT00007334—38150 bp downstream	ISCW023249
Iricinus_Scaffold_13	85,825,000	85,875,000	NA	NA
Iricinus_Scaffold_13	86,250,000	86,300,000	NA	NA
Iricinus_Scaffold_13	86,325,000	86,375,000	NA	NA
Iricinus_Scaffold_13	86,350,000	86,400,000	NA	NA
Iricinus_Scaffold_13	86,375,000	86,425,000	NA	NA
Iricinus_Scaffold_13	86,400,000	86,450,000	NA	NA
Iricinus_Scaffold_13	86,800,000	86,850,000	NA	NA

**Fig. 5:**
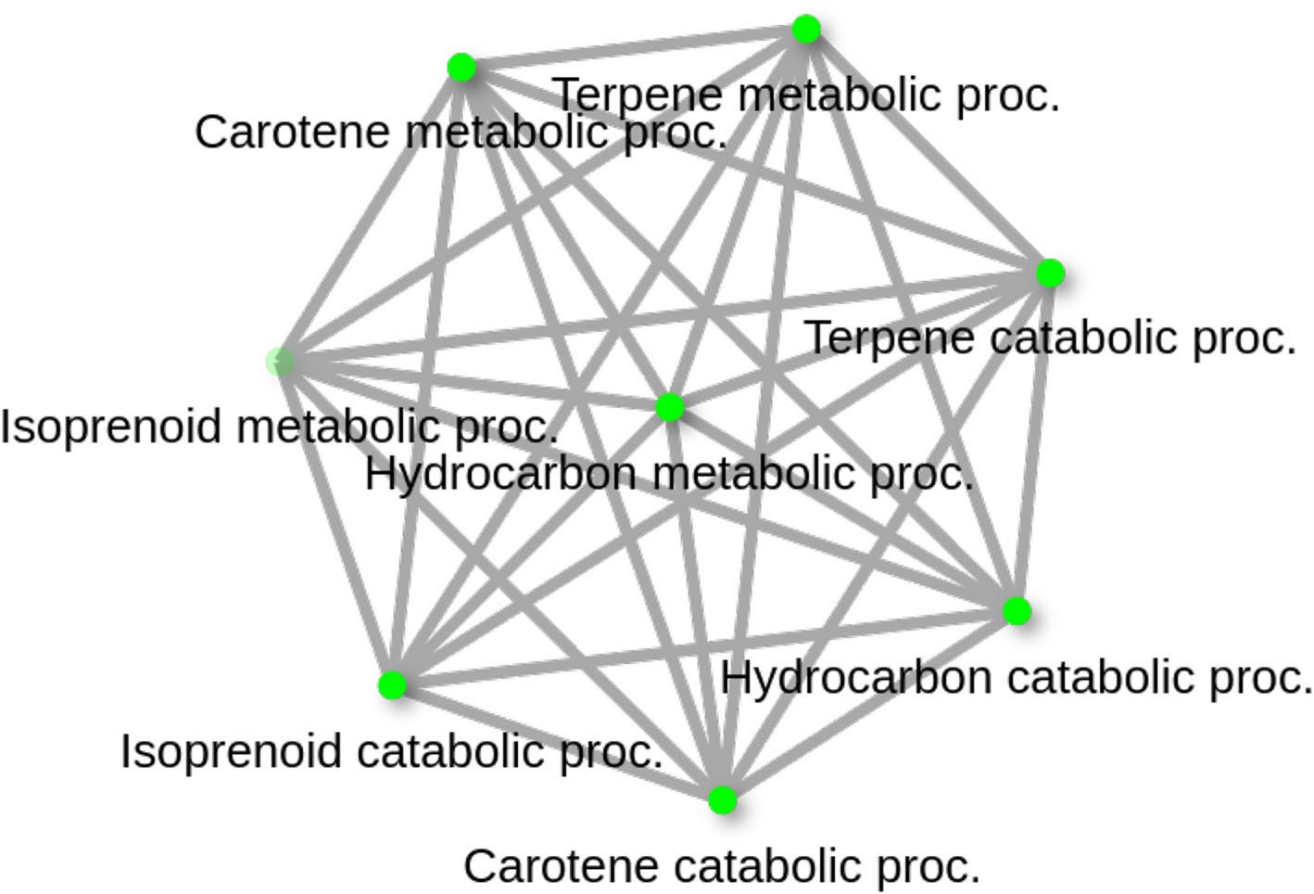
Gene ontology network of significantly enriched Biological Process (BP) terms associated with differentiated genomic regions between Turkish and German *I. ricinus* populations. Nodes represent GO terms inferred from *I. scapularis* homologs; node size reflects the number of associated genes. Edges connect pathways sharing 20% or more genes, with edge thickness indicating the extent of overlap.

Gene ontology enrichment analysis revealed four significantly enriched Biological Process (BP) terms: carotene metabolic process, terpene metabolic process, hydrocarbon metabolic process, and isoprenoid catabolic process (FDR < 0.2, *p* < 0.05; Table 3, Fig. 5).

**Table 3:** Significantly enriched GO Biological Process terms (FDR < 0.2) for genes near highly differentiated genomic windows between Turkish and German *I. ricinus* populations. Results are based on *I. scapularis* homologs and include pathway name, GO ID, and enrichment statistics.

Enrichment FDR	nGenes	Pathway genes	Fold enrichment	Pathways	Go term
2.30E-04	2	3	413.9	Carotene metabolic proc	GO:0016119
2.30E-04	2	3	413.9	Carotene catabolic proc	GO:0016121
2.30E-04	2	3	413.9	Terpene metabolic proc	GO:0042214
2.30E-04	2	3	413.9	Terpene catabolic proc	GO:0046247
2.30E-04	2	3	413.9	Hydrocarbon metabolic proc	GO:0120252
2.30E-04	2	3	413.9	Hydrocarbon catabolic proc	GO:0120253
3.90E-04	2	4	310.4	Isoprenoid catabolic proc	GO:0008300
2.40E-02	2	30	41.4	Isoprenoid metabolic proc	GO:0006720

## Discussion

In this study, we generated whole-genome sequencing data from *I. ricinus* individuals representing four geographically distinct populations across Türkiye. This effort contributes valuable genomic resources for this ecologically and medically important vector species. Future studies integrating pathogen detection could provide further insight into these dynamics. Notably, the sampled populations originate from regions where TBE is not currently endemic, offering a unique genomic perspective that has been largely underrepresented in previous studies. The inclusion of data from these non-endemic areas enhances the geographical and epidemiological scope of existing *I. ricinus* genomic databases and provides a crucial foundation for future comparative and functional genomic analyses aimed at understanding tick adaptation, pathogen-vector interactions, and potential for TBE emergence.

To gain insight into the genetic differentiation and population structure of *I. ricinus*, we employed both PCA and ADMIXTURE. PCA results clearly separated the Turkish, German, and Algerian samples from each other, indicating substantial genetic differentiation. ADMIXTURE analysis further supported this pattern, with distinct ancestry components assigned to Turkish populations compared to those from Germany. Confirming this, previous studies have also highlighted genetic differentiation among geographically distinct populations of various *Ixodes* species. For instance, northern and southern populations of *I. scapularis* in the U.S. showed notable genetic differences^21^, with southern populations exhibiting significantly higher genetic diversity^48^. In line with these findings, it has been shown that *I. ricinus* populations from different regions of the Netherlands exhibited varying susceptibility to local TBEV strains, emphasizing the influence of genetic background on vector competence^17^. Similarly, Liebig et al. (2020) reported higher TBEV infection rates in ticks originating from an endemic area compared to those from a non-endemic region, suggesting the role of local adaptation in shaping tick-virus interactions^18^. Additionally, the observed population structure and admixture patterns among Turkish *I. ricinus* populations (Fig. 2) may reflect underlying ecological differentiation. These regions differ in vegetation, humidity, and possibly host community composition, all of which could influence gene flow and local adaptation in ticks. For example, ORD and KIR are from more forested and humid zones along the Black Sea coast, whereas KAS and ADA lie in more inland or transitional habitats. While we did not directly test ecological variables, the spatial clustering in the PCA and varying admixture proportions support the hypothesis that ecological factors may contribute to genetic structuring. Future work integrating habitat data and host associations will be important to clarify these relationships.

Several studies have attempted to associate genetic differences with vectorial capacity^49–51^. However, identifying specific genetic variants responsible for vectorial capacity remains challenging due to the polygenic nature of vector competence, environmental influences, microbiota, and the complex interactions between vector, host, and pathogen. Therefore, many studies have instead focused on detecting genomic differences between tick populations from disease-endemic and non-endemic regions. For instance, ten DNA variants associated with population differentiation in *I. scapularis* have been identified^48^. Similarly, Cassens et al. (2025) reported clade-specific variants in genes related to xenobiotic detoxification, transmembrane transport, and sulfation—functions that may relate to host use, blood meal processing, and immune evasion. Moreover, a chromosome-level genome assembly of *I. ricinus* revealed expansions in gene families associated with detoxification, lipid metabolism, and chemosensory functions, suggesting functional specialization of certain genomic regions^26^. Our findings parallel these observations. We identified genomic regions with significant diversity differences between Turkish and German *I. ricinus* populations, including genes related to detoxification, lipid metabolism, and chemosensory signaling. This pattern suggests that similar selective pressures—such as local host availability or environmental exposure—may be shaping genomic differentiation in *I. ricinus*. Notably, these pathways are involved in biological processes relevant to vectorial capacity, including host recognition, blood meal digestion, and immune evasion. While our findings reveal intriguing functional patterns, further studies are needed to establish causal links between genomic variation and vector competence. In addition to population-level genomic differentiation, recent studies have shown that genome-wide data can also reveal ecological and microbiological dimensions of tick biology. For example, Baede et al. (2024) and Lesiczka et al. (2025) highlighted horizontal gene transfer and symbiont transmission in *I. ricinus* and *I. inopinatus*, while Krawczyk et al. (2022) reported geographic variation in microbial communities^28–30^. These findings reinforce the broader utility of WGS approaches in understanding vector-associated traits.

Hybridization between *I. ricinus* and other closely related *Ixodes* species is increasingly recognized as a significant contributor to genetic diversity within the genus. Natural hybridization between *I. ricinus* and *I. persulcatus* has been documented in Finland and the Baltic region, providing clear evidence of gene flow between these sympatric species^52,53^. Notably, experimental studies have demonstrated that *I. ricinus* × *I. persulcatus* hybrids retain the ability to acquire and transstadially transmit TBEV, underscoring their potential epidemiological relevance^54^. In light of this, the possible hybridization between *I. ricinus* and *I. inopinatus*—both of which have been reported in Türkiye ^32,55^—emerges as a critical area for further investigation. Exploring this potential hybrid zone could shed light on the mechanisms driving genetic variation and its implications for vector competence and species delimitation within the *I. ricinus* complex.

Host preference is another key factor that may influence the prevalence of diseases transmitted by *Ixodes* ticks. For instance, it has been well documented that northern populations of *I. scapularis* prefer mice as hosts during their immature stages, whereas southern populations prefer lizards^56,57^ and it has been suggested that this variation could be linked to genetic background, which influences host-seeking behaviour^48^. In contrast, *I. ricinus* exhibits remarkable ecological plasticity in its host preferences, which may influence pathogen transmission dynamics in a distinct manner. Rather than displaying geographically structured host specificity, *I. ricinus* maintains a generalist feeding strategy across its range. Larvae and nymphs of this species have been recorded on hosts spanning more than 90 vertebrate families throughout the Palearctic, indicating a high degree of host phylogenetic diversity^4^. Importantly, this host range appears to be driven not by phylogenetic affinity but by ecological opportunity and co-occurrence within shared habitats^4^. Ontogenetic host-switching is also common in *I. ricinus*, with immature stages exploiting different host groups than adults, further enhancing its transmission potential. This supports the idea that *I. ricinus* conforms to the “global generalist but local specialist” paradigm^58^. This flexible host-use strategy not only facilitates the broad geographic distribution of *I. ricinus*, but also may allow local populations to influence pathogen transmission dynamics in a context-dependent manner. This ecological flexibility, while advantageous for the tick, presents substantial challenges for public health surveillance and tick control efforts. Region-specific differences in host availability, habitat conditions, and environmental pressures can lead to local adaptations in behavior or vector competence, as suggested by the genetic structuring and admixture observed among Turkish populations in our study. These findings imply that *I. ricinus* populations may respond differently to environmental and epidemiological drivers depending on their local genomic and ecological context. As such, uniform surveillance or intervention strategies may be insufficient, highlighting the need for localized, data-driven approaches to tick-borne disease monitoring and control.

No ROH segments meeting the defined detection thresholds were identified across Turkish *Ixodes ricinus* populations. This absence of detectable ROH likely reflects extremely high genomic heterozygosity and the rarity of extended homozygous regions. Several factors may contribute to this pattern, including large effective population sizes, high levels of gene flow, and frequent host-mediated dispersal, all of which promote genetic mixing and limit the accumulation of homozygous tracts. The ecological behavior of *I. ricinus*, characterized by generalist host use and broad geographic mobility, is consistent with these observations. Supporting this interpretation, a recent comparative genomic study on *I. scapularis* demonstrated that ticks from Texas, which displayed higher genetic diversity, exhibited fewer and shorter ROH segments compared to ticks from Minnesota and Pennsylvania, where ROH segments were longer and more frequent^49^. Thus, the absence of extended homozygosity in Turkish *I. ricinus* populations likely indicates the maintenance of substantial genetic diversity over time through extensive gene flow and demographic stability. Nevertheless, it should be noted that technical factors such as low variant density, stringent ROH detection thresholds, and variable sequencing depth across scaffolds could also reduce the power to detect ROH segments, particularly shorter tracts. While the analytical parameters were carefully selected to balance sensitivity and specificity, technical limitations cannot be entirely excluded as contributing factors to the observed pattern.

## Conclusions

This study provides new genome-wide data from *I. ricinus* populations in Türkiye, expanding the geographic and epidemiological scope of existing genomic resources for this important vector species. By comparing populations from both TBE-endemic (Germany) and non-endemic (Türkiye) regions, our findings contribute to a broader understanding of tick population structure and genetic diversity across distinct ecological contexts. While the study primarily focuses on TBE due to its recognized association with *I. ricinus* in Europe, it is worth noting that this species also plays a major role in the transmission of Lyme borreliosis. The relatively low incidence of Lyme disease in Türkiye may reflect not only ecological differences but also genetic factors influencing vector competence.

Importantly, the inclusion of non-endemic Turkish populations offers a previously underrepresented genomic perspective that can support future efforts to investigate vector-pathogen interactions and local adaptation. Moreover, this dataset provides a foundation for enhanced tick surveillance at both local and regional scales. Genome-wide data can help detect shifts in population structure, monitor the spread of specific lineages, and—when integrated with pathogen and symbiont profiles—support early warning systems for the emergence or expansion of tick-borne diseases.

## Supplementary Information

Supplementary Information.

## Data Availability

All raw sequence data generated from the whole genome sequencing are available under GenBank BioProject accession number PRJNA1273614.
