# Supplementary material for: Genome-wide insights into genetic diversity of endemic and non-endemic Ixodes ricinus populations
Source: Sci Rep. 2025 Oct 1;15:34199. doi: 10.1038/s41598-025-15640-2 (PMC12489033; doi:10.1038/s41598-025-15640-2)
Supplement: Supplementary file 1 — Supplementary Information. [file 41598_2025_15640_MOESM1_ESM.docx]

**Supplementary Tables**

**Supplementary Table 1.** Sampling information of *I. ricinus* individuals collected in Türkiye.

| **Nr** | **Population** | **Abbreviation** | **Province** | **Sample ID** | **Coordinate** | **Method/ Host** |
| --- | --- | --- | --- | --- | --- | --- |
| 1 | Marmara | ADA | Adapazarı | ADA30 | 40.77763 °N; 30.62295 °E | Cow |
| 2 | Marmara | ADA | Adapazarı | ADA35-1 | 40.77763 °N; 30.62295 °E | Cow |
| 3 | Marmara | ADA | Adapazarı | ADA35-2 | 40.77763 °N; 30.62295 °E | Cow |
| 4 | Marmara | ADA | Adapazarı | ADA35-3 | 40.77763 °N; 30.62295 °E | Cow |
| 5 | Western Black Sea | KAS | Kastamonu | KAS18-3b | 41.14138 °N; 33.7777 °E | Cow |
| 6 | Western Black Sea | KAS | Kastamonu | KAS17-2 | 41.66417 °N, 33.13889 °E | Flagging |
| 7 | Western Black Sea | KAS | Kastamonu | KAS18-14 | 41.45694 °N; 33.40777 °E | Cow |
| 8 | Northwest/Thrace | KIR | Kırklareli | KIR21-2a | 41.66995 °N; 28.05138 °E | Dog |
| 9 | Northwest/Thrace | KIR | Kırklareli | KIR21-2b | 41.66995 °N; 28.05138 °E | Dog |
| 10 | Northwest/Thrace | KIR | Kırklareli | KIR21-6 | 41.66995 °N; 28.05138 °E | Dog |
| 11 | Northwest/Thrace | KIR | Kırklareli | KIRK83 | 41.87194 °N; 27.80999 °E | Flagging |
| 12 | Northwest/Thrace | KIR | Kırklareli | KIRK82 | 41.87194 °N; 27.80999 °E | Flagging |
| 13 | Central Black Sea | ORD | Ordu | ORD18-4 | 40.88418°N; 37.77249 °E | Cow |
| 14 | Central Black Sea | ORD | Ordu | ORD18_61 | 40.51972°N; 37.78954 °E | Cow |
| 15 | Central Black Sea | ORD | Ordu | ORD18-2 | 40.88418°N; 37.77249 °E | Cow |

**Supplementary Table 2.** Detailed information on sequencing and alignment for each sample

| **Population** | **Sample ID** | **Total_Reads** | **Mapped_reads** | **Paired&Mated** | **Properly_Aligned** | **Percent_Aligned** |
| --- | --- | --- | --- | --- | --- | --- |
| ADA | ADA30 | 250819948 | 233920183 | 230621351 | 184902796 | 93,26 |
| ADA | ADA35_1 | 226971378 | 212445783 | 208506959 | 165699679 | 93 |
| ADA | ADA35_2 | 272415256 | 247248351 | 242831004 | 194346535 | 90 |
| ADA | ADA35_3 | 233435133 | 218215398 | 214265932 | 170300841 | 93 |
| KAS | KAS17_2 | 151749088 | 148473919 | 147136996 | 120590224 | 97 |
| KAS | KAS18_14 | 235400791 | 170662182 | 167164089 | 130565149 | 72,5 |
| KAS | KAS18_3b | 217883381 | 207986712 | 203262415 | 163574583 | 95 |
| KIR | KIR21_2a | 208323871 | 199544841 | 196089425 | 157167125 | 95 |
| KIR | KIR21_2b | 299730002 | 287637088 | 284354517 | 231121425 | 95 |
| KIR | KIR21_6 | 230693094 | 220649235 | 216471394 | 173509297 | 95 |
| KIR | KIRK82 | 238901737 | 233573613 | 230589349 | 183155274 | 97 |
| KIR | KIRK83 | 244202177 | 238952187 | 236100367 | 184360494 | 97 |
| ORD | ORD18_2 | 301602681 | 292969523 | 289541229 | 232379647 | 97 |
| ORD | ORD18_4 | 183128225 | 178869041 | 176793878 | 139921791 | 97,67 |
| ORD | ORD18_61 | 212002950 | 199482829 | 197228593 | 154831725 | 94 |
| GER | SRR23586399 | 1234259269 | 961685710 | 955562572 | 760016593 | 77,92 |
| GER | SRR23586400 | 999013347 | 573296962 | 567980143 | 497685710 | 57,39 |
| GER | SRR23586401 | 1044950570 | 741832842 | 736367978 | 653981853 | 70,99 |
| GER | SRR23586402 | 1088528022 | 1016260243 | 1009776454 | 863664961 | 79,34 |
| GER | SRR23586403 | 1090855302 | 1029784990 | 1090855302 | 865682622 | 79,36 |
| GER | SRR23586404 | 1186972497 | 1171662292 | 1186972497 | 981968208 | 82,73 |
| ALG | SRR23586405 | 62406195 | 61512319 | 62406195 | 51359327 | 82,3 |
| ALG | SRR23586406 | 521697539 | 512152434 | 505076932 | 425350106 | 98,17 |

**Supplementary Table 3.** Mean differences in nucleotide diversity between Turkish and German populations across all 14 major scaffolds, along with their significance based on FDR-adjusted p-values

| Chr | Pop | Mean_Diff_tP | P_value | FDR |
| --- | --- | --- | --- | --- |
| Scaffold_1 | ADA | 0.0091 | 2.16E-94 | 1.21E-92 |
|  | KAS | 0.0056 | 2.27E-44 | 1.82E-43 |
|  | KIR | 0.0063 | 1.29E-54 | 1.44E-53 |
|  | ORD | 0.0066 | 1.76E-55 | 2.46E-54 |
| Scaffold_2 | ADA | 0.0038 | 4.24E-19 | 1.08E-18 |
|  | KAS | 0.0052 | 1.19E-33 | 5.13E-33 |
|  | KIR | 0.0037 | 7.01E-18 | 1.71E-17 |
|  | ORD | 0.0062 | 1.71E-43 | 1.06E-42 |
| Scaffold_3 | ADA | 0.0035 | 1.07E-07 | 1.46E-07 |
|  | KAS | 0.0051 | 3.65E-14 | 6.39E-14 |
|  | KIR | 0.0031 | 1.90E-06 | 2.41E-06 |
|  | ORD | 0.0053 | 2.71E-15 | 5.43E-15 |
| Scaffold_4 | ADA | 0.0067 | 1.25E-43 | 8.75E-43 |
|  | KAS | 0.0084 | 5.06E-65 | 1.42E-63 |
|  | KIR | 0.0062 | 4.56E-41 | 2.55E-40 |
|  | ORD | 0.0078 | 3.07E-61 | 5.74E-60 |
| Scaffold_5 | ADA | 0.0047 | 5.39E-28 | 1.89E-27 |
|  | KAS | 0.0054 | 1.99E-35 | 9.29E-35 |
|  | KIR | 0.0043 | 5.42E-24 | 1.69E-23 |
|  | ORD | 0.0065 | 3.25E-49 | 3.03E-48 |
| Scaffold_6 | ADA | 0.0034 | 1.40E-15 | 2.91E-15 |
|  | KAS | 0.0044 | 1.59E-24 | 5.25E-24 |
|  | KIR | 0.0035 | 3.38E-16 | 7.57E-16 |
|  | ORD | 0.0057 | 1.45E-39 | 7.38E-39 |
| Scaffold_7 | ADA | 0.0029 | 4.34E-05 | 4.96E-05 |
|  | KAS | 0.0051 | 7.93E-12 | 1.27E-11 |
|  | KIR | 0.0024 | 9.48E-04 | 0.0010 |
|  | ORD | 0.0056 | 8.33E-15 | 1.55E-14 |
| Scaffold_8 | ADA | 0.0012 | 0.0131 | 0.0136 |
|  | KAS | 0.0038 | 2.50E-14 | 4.52E-14 |
|  | KIR | 0.0014 | 0.0067 | 0.0070 |
|  | ORD | 0.0039 | 3.82E-14 | 6.48E-14 |
| Scaffold_9 | ADA | 0.0036 | 1.17E-06 | 1.52E-06 |
|  | KAS | 0.0052 | 1.84E-11 | 2.86E-11 |
|  | KIR | 0.0029 | 1.11E-04 | 1.24E-04 |
|  | ORD | 0.0049 | 9.49E-11 | 1.44E-10 |
| Scaffold_10 | ADA | 0.0027 | 4.26E-09 | 6.12E-09 |
|  | KAS | 0.0038 | 6.49E-16 | 1.40E-15 |
|  | KIR | 0.0025 | 1.15E-07 | 1.53E-07 |
|  | ORD | 0.0053 | 3.00E-28 | 1.12E-27 |
| Scaffold_11 | ADA | 0.0061 | 4.00E-19 | 1.07E-18 |
|  | KAS | 0.0055 | 8.21E-17 | 1.92E-16 |
|  | KIR | 0.0053 | 3.05E-15 | 5.90E-15 |
|  | ORD | 0.0077 | 2.23E-29 | 8.94E-29 |
| Scaffold_12 | ADA | 0.0000 | 0.9356 | 0.9356 |
|  | KAS | 0.0013 | 3.72E-06 | 4.62E-06 |
|  | KIR | -0.0004 | 0.1305 | 0.1329 |
|  | ORD | 0.0026 | 5.59E-21 | 1.65E-20 |
| Scaffold_13 | ADA | 0.0034 | 3.92E-05 | 4.57E-05 |
|  | KAS | 0.0050 | 3.85E-09 | 5.67E-09 |
|  | KIR | 0.0028 | 6.06E-04 | 6.66E-04 |
|  | ORD | 0.0057 | 2.11E-12 | 3.47E-12 |
| Scaffold_14 | ADA | 0.0035 | 1.12E-05 | 1.34E-05 |
|  | KAS | 0.0043 | 5.89E-08 | 8.25E-08 |
|  | KIR | 0.0036 | 5.51E-06 | 6.71E-06 |
|  | ORD | 0.0077 | 1.31E-19 | 3.67E-19 |

**Supplementary Table 4.** Genomic windows showing significant differences in nucleotide diversity (Theta Pi) between the ADA and GER populations of *I. ricinus* across all 14 major scaffolds.

| **Pop** | **Chr** | **Pos** | **tP** | **nSites** | **Ger_tP** | **Diff_tP** | **Z_score** | **P_value** |
| --- | --- | --- | --- | --- | --- | --- | --- | --- |
| ADA | Scaffold_1 | 40275000 | 0.0575 | 14419 | 0.0197 | 0.0378 | 4.6482 | 3.35E-06 |
| ADA | Scaffold_1 | 100225000 | 0.0410 | 4409 | 0.0647 | -0.0238 | -4.5538 | 5.27E-06 |
| ADA | Scaffold_1 | 114125000 | 0.0568 | 10546 | 0.0096 | 0.0472 | 6.0551 | 1.40E-09 |
| ADA | Scaffold_1 | 114150000 | 0.0606 | 9862 | 0.0125 | 0.0481 | 6.1939 | 5.87E-10 |
| ADA | Scaffold_1 | 122275000 | 0.0603 | 3719 | 0.0234 | 0.0369 | 4.5154 | 6.32E-06 |
| ADA | Scaffold_1 | 122300000 | 0.0622 | 3515 | 0.0212 | 0.0410 | 5.1264 | 2.95E-07 |
| ADA | Scaffold_1 | 136150000 | 0.0719 | 5966 | 0.0336 | 0.0383 | 4.7257 | 2.29E-06 |
| ADA | Scaffold_1 | 136175000 | 0.0662 | 7850 | 0.0261 | 0.0402 | 5.0072 | 5.52E-07 |
| ADA | Scaffold_1 | 136200000 | 0.0581 | 4872 | 0.0207 | 0.0374 | 4.5856 | 4.53E-06 |
| ADA | Scaffold_1 | 139275000 | 0.0706 | 9799 | 0.0323 | 0.0383 | 4.7292 | 2.25E-06 |
| ADA | Scaffold_1 | 158000000 | 0.0702 | 6111 | 0.0217 | 0.0485 | 6.2513 | 4.07E-10 |
| ADA | Scaffold_1 | 158025000 | 0.0658 | 15094 | 0.0244 | 0.0414 | 5.1940 | 2.06E-07 |
| ADA | Scaffold_1 | 170075000 | 0.0520 | 2988 | 0.0118 | 0.0402 | 5.0059 | 5.56E-07 |
| ADA | Scaffold_1 | 170100000 | 0.0541 | 5416 | 0.0160 | 0.0381 | 4.6949 | 2.67E-06 |
| ADA | Scaffold_1 | 180050000 | 0.0660 | 6715 | 0.0254 | 0.0407 | 5.0824 | 3.73E-07 |
| ADA | Scaffold_1 | 180075000 | 0.0616 | 9909 | 0.0239 | 0.0377 | 4.6379 | 3.52E-06 |
| ADA | Scaffold_1 | 180125000 | 0.0652 | 11452 | 0.0246 | 0.0407 | 5.0781 | 3.81E-07 |
| ADA | Scaffold_1 | 180150000 | 0.0733 | 16464 | 0.0219 | 0.0514 | 6.6859 | 2.30E-11 |
| ADA | Scaffold_1 | 180175000 | 0.0704 | 13124 | 0.0245 | 0.0458 | 5.8495 | 4.93E-09 |
| ADA | Scaffold_1 | 180275000 | 0.0800 | 6038 | 0.0297 | 0.0502 | 6.5109 | 7.47E-11 |
| ADA | Scaffold_1 | 180300000 | 0.0607 | 2235 | 0.0214 | 0.0393 | 4.8804 | 1.06E-06 |
| ADA | Scaffold_2 | 36675000 | 0.0181 | 18496 | 0.0470 | -0.0289 | -4.5684 | 4.91E-06 |
| ADA | Scaffold_2 | 36700000 | 0.0141 | 16376 | 0.0460 | -0.0319 | -5.0003 | 5.72E-07 |
| ADA | Scaffold_2 | 44700000 | 0.0477 | 6105 | 0.0111 | 0.0366 | 4.6085 | 4.06E-06 |
| ADA | Scaffold_2 | 94850000 | 0.0713 | 1421 | 0.0200 | 0.0513 | 6.6646 | 2.65E-11 |
| ADA | Scaffold_2 | 129475000 | 0.0338 | 1304 | 0.0626 | -0.0288 | -4.5596 | 5.12E-06 |
| ADA | Scaffold_3 | 32475000 | 0.0816 | 8346 | 0.0537 | 0.0279 | 4.5533 | 5.28E-06 |
| ADA | Scaffold_3 | 35325000 | 0.0740 | 8454 | 0.0470 | 0.0270 | 4.3810 | 1.18E-05 |
| ADA | Scaffold_3 | 71550000 | 0.0338 | 1101 | 0.0558 | -0.0220 | -4.5285 | 5.94E-06 |
| ADA | Scaffold_3 | 92775000 | 0.0444 | 4824 | 0.0155 | 0.0289 | 4.7351 | 2.19E-06 |
| ADA | Scaffold_3 | 92800000 | 0.0478 | 3475 | 0.0157 | 0.0321 | 5.3167 | 1.06E-07 |
| ADA | Scaffold_3 | 140175000 | 0.1068 | 1415 | 0.0788 | 0.0281 | 4.5785 | 4.68E-06 |
| ADA | Scaffold_3 | 140350000 | 0.1006 | 2796 | 0.0718 | 0.0287 | 4.7026 | 2.57E-06 |
| ADA | Scaffold_3 | 140775000 | 0.1115 | 1822 | 0.0824 | 0.0291 | 4.7670 | 1.87E-06 |
| ADA | Scaffold_3 | 140800000 | 0.1135 | 1758 | 0.0836 | 0.0299 | 4.9201 | 8.65E-07 |
| ADA | Scaffold_3 | 140850000 | 0.1018 | 2014 | 0.0691 | 0.0327 | 5.4150 | 6.13E-08 |
| ADA | Scaffold_3 | 140875000 | 0.1034 | 2075 | 0.0745 | 0.0289 | 4.7286 | 2.26E-06 |
| ADA | Scaffold_4 | 37475000 | 0.0751 | 2251 | 0.0365 | 0.0386 | 4.6728 | 2.97E-06 |
| ADA | Scaffold_4 | 54125000 | 0.0666 | 8688 | 0.0255 | 0.0411 | 5.0387 | 4.69E-07 |
| ADA | Scaffold_4 | 80600000 | 0.0779 | 4990 | 0.0297 | 0.0482 | 6.0674 | 1.30E-09 |
| ADA | Scaffold_4 | 80625000 | 0.0839 | 6006 | 0.0380 | 0.0459 | 5.7384 | 9.56E-09 |
| ADA | Scaffold_4 | 111400000 | 0.0672 | 5475 | 0.0210 | 0.0462 | 5.7751 | 7.69E-09 |
| ADA | Scaffold_4 | 111425000 | 0.0693 | 11750 | 0.0261 | 0.0432 | 5.3429 | 9.15E-08 |
| ADA | Scaffold_4 | 165700000 | 0.0625 | 7084 | 0.0207 | 0.0419 | 5.1478 | 2.64E-07 |
| ADA | Scaffold_4 | 165725000 | 0.0653 | 9941 | 0.0247 | 0.0406 | 4.9705 | 6.68E-07 |
| ADA | Scaffold_4 | 165750000 | 0.0606 | 9428 | 0.0226 | 0.0380 | 4.5877 | 4.48E-06 |
| ADA | Scaffold_4 | 165775000 | 0.0589 | 9750 | 0.0207 | 0.0382 | 4.6193 | 3.85E-06 |
| ADA | Scaffold_4 | 165800000 | 0.0618 | 5266 | 0.0227 | 0.0391 | 4.7473 | 2.06E-06 |
| ADA | Scaffold_4 | 165925000 | 0.0607 | 3245 | 0.0227 | 0.0381 | 4.5972 | 4.28E-06 |
| ADA | Scaffold_5 | 53675000 | 0.0488 | 1060 | 0.0080 | 0.0408 | 5.4420 | 5.27E-08 |
| ADA | Scaffold_5 | 60125000 | 0.0842 | 1167 | 0.0491 | 0.0351 | 4.5918 | 4.39E-06 |
| ADA | Scaffold_5 | 86175000 | 0.0707 | 1060 | 0.0306 | 0.0401 | 5.3399 | 9.30E-08 |
| ADA | Scaffold_5 | 120625000 | 0.0582 | 2850 | 0.0103 | 0.0479 | 6.4952 | 8.29E-11 |
| ADA | Scaffold_5 | 120650000 | 0.0567 | 2919 | 0.0104 | 0.0463 | 6.2541 | 4.00E-10 |
| ADA | Scaffold_6 | 96025000 | 0.0732 | 3108 | 0.0121 | 0.0611 | 8.6903 | 3.62E-18 |
| ADA | Scaffold_6 | 96050000 | 0.0692 | 5831 | 0.0156 | 0.0536 | 7.5647 | 3.89E-14 |
| ADA | Scaffold_6 | 159325000 | 0.0656 | 6585 | 0.0317 | 0.0339 | 4.5890 | 4.45E-06 |
| ADA | Scaffold_6 | 159700000 | 0.0616 | 2558 | 0.0196 | 0.0419 | 5.8022 | 6.55E-09 |
| ADA | Scaffold_6 | 159725000 | 0.0571 | 5279 | 0.0215 | 0.0356 | 4.8398 | 1.30E-06 |
| ADA | Scaffold_6 | 159850000 | 0.0639 | 8036 | 0.0297 | 0.0342 | 4.6372 | 3.53E-06 |
| ADA | Scaffold_6 | 159925000 | 0.0581 | 6373 | 0.0211 | 0.0370 | 5.0568 | 4.26E-07 |
| ADA | Scaffold_6 | 159950000 | 0.0581 | 6149 | 0.0222 | 0.0358 | 4.8793 | 1.06E-06 |
| ADA | Scaffold_7 | 24075000 | 0.0631 | 1414 | 0.0336 | 0.0295 | 5.5367 | 3.08E-08 |
| ADA | Scaffold_7 | 24100000 | 0.0580 | 1457 | 0.0281 | 0.0300 | 5.6314 | 1.79E-08 |
| ADA | Scaffold_7 | 55425000 | 0.0465 | 1762 | 0.0720 | -0.0255 | -5.6980 | 1.21E-08 |
| ADA | Scaffold_7 | 55450000 | 0.0506 | 1440 | 0.0752 | -0.0246 | -5.5158 | 3.47E-08 |
| ADA | Scaffold_7 | 102775000 | 0.0409 | 5292 | 0.0670 | -0.0261 | -5.8343 | 5.40E-09 |
| ADA | Scaffold_7 | 102800000 | 0.0456 | 3598 | 0.0791 | -0.0335 | -7.3300 | 2.30E-13 |
| ADA | Scaffold_7 | 105275000 | 0.0619 | 1823 | 0.0372 | 0.0247 | 4.5551 | 5.24E-06 |
| ADA | Scaffold_7 | 105300000 | 0.0625 | 1269 | 0.0370 | 0.0255 | 4.7103 | 2.47E-06 |
| ADA | Scaffold_7 | 106075000 | 0.0744 | 1526 | 0.0438 | 0.0307 | 5.7700 | 7.93E-09 |
| ADA | Scaffold_7 | 106100000 | 0.0762 | 15326 | 0.0459 | 0.0303 | 5.6983 | 1.21E-08 |
| ADA | Scaffold_7 | 106125000 | 0.0766 | 22731 | 0.0476 | 0.0290 | 5.4287 | 5.68E-08 |
| ADA | Scaffold_7 | 106150000 | 0.0765 | 14436 | 0.0488 | 0.0276 | 5.1531 | 2.56E-07 |
| ADA | Scaffold_7 | 106175000 | 0.0740 | 6594 | 0.0463 | 0.0276 | 5.1549 | 2.54E-07 |
| ADA | Scaffold_7 | 113125000 | 0.0239 | 25318 | 0.0462 | -0.0223 | -5.0496 | 4.43E-07 |
| ADA | Scaffold_7 | 113525000 | 0.0540 | 1002 | 0.0261 | 0.0279 | 5.2093 | 1.90E-07 |
| ADA | Scaffold_8 | 2575000 | 0.0508 | 1030 | 0.0787 | -0.0280 | -5.5321 | 3.16E-08 |
| ADA | Scaffold_8 | 2600000 | 0.0517 | 1030 | 0.0796 | -0.0279 | -5.5252 | 3.29E-08 |
| ADA | Scaffold_8 | 33125000 | 0.0699 | 2473 | 0.0407 | 0.0293 | 5.3097 | 1.10E-07 |
| ADA | Scaffold_8 | 33150000 | 0.0699 | 2473 | 0.0407 | 0.0293 | 5.3097 | 1.10E-07 |
| ADA | Scaffold_8 | 44550000 | 0.0271 | 1141 | 0.0577 | -0.0306 | -6.0271 | 1.67E-09 |
| ADA | Scaffold_8 | 70550000 | 0.0481 | 2848 | 0.0164 | 0.0317 | 5.7730 | 7.79E-09 |
| ADA | Scaffold_8 | 70575000 | 0.0552 | 2676 | 0.0196 | 0.0356 | 6.5200 | 7.03E-11 |
| ADA | Scaffold_8 | 71250000 | 0.0619 | 9324 | 0.0366 | 0.0253 | 4.5521 | 5.31E-06 |
| ADA | Scaffold_8 | 71275000 | 0.0767 | 8731 | 0.0483 | 0.0284 | 5.1491 | 2.62E-07 |
| ADA | Scaffold_8 | 111825000 | 0.0425 | 3041 | 0.0653 | -0.0227 | -4.5398 | 5.63E-06 |
| ADA | Scaffold_9 | 38350000 | 0.0136 | 5605 | 0.0354 | -0.0218 | -4.4371 | 9.12E-06 |
| ADA | Scaffold_9 | 75425000 | 0.0676 | 2074 | 0.0344 | 0.0332 | 5.4585 | 4.80E-08 |
| ADA | Scaffold_10 | 50200000 | 0.0499 | 4788 | 0.0262 | 0.0237 | 4.5662 | 4.97E-06 |
| ADA | Scaffold_10 | 63950000 | 0.0651 | 2382 | 0.0373 | 0.0278 | 5.4273 | 5.72E-08 |
| ADA | Scaffold_10 | 63975000 | 0.0628 | 2833 | 0.0391 | 0.0238 | 4.5718 | 4.84E-06 |
| ADA | Scaffold_10 | 81275000 | 0.0529 | 2881 | 0.0283 | 0.0246 | 4.7437 | 2.10E-06 |
| ADA | Scaffold_10 | 81300000 | 0.0661 | 1867 | 0.0269 | 0.0392 | 7.8399 | 4.51E-15 |
| ADA | Scaffold_10 | 101025000 | 0.0480 | 2801 | 0.0221 | 0.0259 | 5.0188 | 5.20E-07 |
| ADA | Scaffold_11 | 56575000 | 0.1019 | 1270 | 0.0617 | 0.0402 | 5.0848 | 3.68E-07 |
| ADA | Scaffold_12 | 6125000 | 0.0344 | 1530 | 0.0494 | -0.0150 | -4.9239 | 8.48E-07 |
| ADA | Scaffold_12 | 15925000 | 0.0296 | 1814 | 0.0439 | -0.0143 | -4.6921 | 2.70E-06 |
| ADA | Scaffold_12 | 24150000 | 0.0657 | 1913 | 0.0487 | 0.0171 | 5.9358 | 2.92E-09 |
| ADA | Scaffold_12 | 26400000 | 0.0103 | 7329 | 0.0274 | -0.0172 | -5.6523 | 1.58E-08 |
| ADA | Scaffold_12 | 26425000 | 0.0141 | 5737 | 0.0383 | -0.0243 | -8.0545 | 7.98E-16 |
| ADA | Scaffold_12 | 26450000 | 0.0150 | 2814 | 0.0284 | -0.0134 | -4.3886 | 1.14E-05 |
| ADA | Scaffold_12 | 52275000 | 0.0887 | 2321 | 0.0734 | 0.0153 | 5.3317 | 9.73E-08 |
| ADA | Scaffold_12 | 102250000 | 0.0233 | 1298 | 0.0368 | -0.0136 | -4.4369 | 9.12E-06 |
| ADA | Scaffold_12 | 108875000 | 0.0612 | 1076 | 0.0479 | 0.0133 | 4.6538 | 3.26E-06 |
| ADA | Scaffold_13 | 78325000 | 0.0789 | 2443 | 0.0444 | 0.0345 | 4.4476 | 8.69E-06 |
| ADA | Scaffold_13 | 83300000 | 0.0762 | 2801 | 0.0349 | 0.0413 | 5.4064 | 6.43E-08 |
| ADA | Scaffold_13 | 84825000 | 0.0872 | 2814 | 0.0504 | 0.0368 | 4.7660 | 1.88E-06 |
| ADA | Scaffold_13 | 84975000 | 0.0762 | 3787 | 0.0383 | 0.0379 | 4.9229 | 8.53E-07 |
| ADA | Scaffold_13 | 85000000 | 0.0804 | 4688 | 0.0415 | 0.0389 | 5.0623 | 4.14E-07 |
| ADA | Scaffold_13 | 85025000 | 0.0889 | 1818 | 0.0424 | 0.0464 | 6.1258 | 9.02E-10 |
| ADA | Scaffold_13 | 85050000 | 0.0657 | 1705 | 0.0276 | 0.0381 | 4.9526 | 7.32E-07 |
| ADA | Scaffold_13 | 85575000 | 0.0895 | 3553 | 0.0530 | 0.0365 | 4.7284 | 2.26E-06 |
| ADA | Scaffold_13 | 85600000 | 0.0912 | 3008 | 0.0558 | 0.0354 | 4.5728 | 4.81E-06 |
| ADA | Scaffold_13 | 85850000 | 0.0934 | 1000 | 0.0545 | 0.0389 | 5.0594 | 4.21E-07 |
| ADA | Scaffold_13 | 86100000 | 0.0938 | 2527 | 0.0557 | 0.0381 | 4.9455 | 7.59E-07 |
| ADA | Scaffold_13 | 86125000 | 0.0762 | 1187 | 0.0423 | 0.0339 | 4.3562 | 1.32E-05 |
| ADA | Scaffold_13 | 86250000 | 0.0757 | 2006 | 0.0393 | 0.0365 | 4.7215 | 2.34E-06 |
| ADA | Scaffold_13 | 86275000 | 0.0804 | 1411 | 0.0327 | 0.0477 | 6.3005 | 2.97E-10 |
| ADA | Scaffold_13 | 86350000 | 0.0840 | 3082 | 0.0417 | 0.0423 | 5.5428 | 2.98E-08 |
| ADA | Scaffold_13 | 86375000 | 0.0929 | 5343 | 0.0563 | 0.0366 | 4.7343 | 2.20E-06 |
| ADA | Scaffold_13 | 86400000 | 0.0982 | 10932 | 0.0565 | 0.0417 | 5.4506 | 5.02E-08 |
| ADA | Scaffold_13 | 86425000 | 0.0967 | 10467 | 0.0521 | 0.0447 | 5.8742 | 4.25E-09 |
| ADA | Scaffold_13 | 86825000 | 0.0886 | 2009 | 0.0473 | 0.0413 | 5.3995 | 6.68E-08 |
| ADA | Scaffold_14 | 46225000 | 0.0424 | 1771 | 0.0100 | 0.0323 | 4.9503 | 7.41E-07 |
| ADA | Scaffold_14 | 48050000 | 0.0412 | 3566 | 0.0107 | 0.0306 | 4.6458 | 3.39E-06 |
| ADA | Scaffold_14 | 48075000 | 0.0377 | 4617 | 0.0068 | 0.0309 | 4.7087 | 2.49E-06 |
| ADA | Scaffold_14 | 55750000 | 0.0737 | 8687 | 0.0404 | 0.0333 | 5.1122 | 3.18E-07 |
| ADA | Scaffold_14 | 55775000 | 0.0641 | 11984 | 0.0331 | 0.0310 | 4.7187 | 2.37E-06 |
| ADA | Scaffold_14 | 55825000 | 0.0689 | 11663 | 0.0376 | 0.0313 | 4.7762 | 1.79E-06 |

**Supplementary Table 5.** Genomic windows showing significant differences in nucleotide diversity (Theta Pi) between the KAS and GER populations of *I. ricinus* across all 14 major scaffolds.

| **Pop** | **Chr** | **Pos** | **tP** | **nSites** | **Ger_tP** | **Diff_tP** | **Z_score** | **P_value** |
| --- | --- | --- | --- | --- | --- | --- | --- | --- |
| KAS | Scaffold_1 | 40275000 | 0.0613 | 14419 | 0.0197 | 0.0416 | 5.1708 | 2.33E-07 |
| KAS | Scaffold_1 | 42950000 | 0.0743 | 1470 | 0.0343 | 0.0400 | 4.9348 | 8.02E-07 |
| KAS | Scaffold_1 | 114125000 | 0.0555 | 10546 | 0.0096 | 0.0459 | 5.7909 | 7.00E-09 |
| KAS | Scaffold_1 | 114150000 | 0.0630 | 9862 | 0.0125 | 0.0505 | 6.4515 | 1.11E-10 |
| KAS | Scaffold_1 | 158000000 | 0.0783 | 6111 | 0.0217 | 0.0566 | 7.3329 | 2.25E-13 |
| KAS | Scaffold_1 | 158025000 | 0.0698 | 15094 | 0.0244 | 0.0455 | 5.7257 | 1.03E-08 |
| KAS | Scaffold_1 | 166250000 | 0.0566 | 1075 | 0.0180 | 0.0386 | 4.7364 | 2.18E-06 |
| KAS | Scaffold_1 | 170075000 | 0.0714 | 2988 | 0.0118 | 0.0596 | 7.7712 | 7.78E-15 |
| KAS | Scaffold_1 | 170100000 | 0.0673 | 5416 | 0.0160 | 0.0512 | 6.5584 | 5.44E-11 |
| KAS | Scaffold_1 | 170125000 | 0.0601 | 11448 | 0.0189 | 0.0412 | 5.1041 | 3.32E-07 |
| KAS | Scaffold_1 | 170150000 | 0.0598 | 14268 | 0.0196 | 0.0402 | 4.9663 | 6.83E-07 |
| KAS | Scaffold_1 | 180125000 | 0.0651 | 11452 | 0.0246 | 0.0406 | 5.0161 | 5.27E-07 |
| KAS | Scaffold_1 | 180150000 | 0.0734 | 16464 | 0.0219 | 0.0515 | 6.6024 | 4.05E-11 |
| KAS | Scaffold_1 | 180175000 | 0.0693 | 13124 | 0.0245 | 0.0448 | 5.6282 | 1.82E-08 |
| KAS | Scaffold_1 | 180275000 | 0.0806 | 6038 | 0.0297 | 0.0508 | 6.5010 | 7.98E-11 |
| KAS | Scaffold_1 | 184050000 | 0.0574 | 1568 | 0.0195 | 0.0379 | 4.6350 | 3.57E-06 |
| KAS | Scaffold_2 | 21325000 | 0.0612 | 4653 | 0.0222 | 0.0391 | 4.7714 | 1.83E-06 |
| KAS | Scaffold_2 | 87150000 | 0.0842 | 13182 | 0.0459 | 0.0383 | 4.6618 | 3.13E-06 |
| KAS | Scaffold_2 | 92400000 | 0.0473 | 1538 | 0.0101 | 0.0372 | 4.5121 | 6.42E-06 |
| KAS | Scaffold_2 | 94850000 | 0.0812 | 1421 | 0.0200 | 0.0613 | 7.9186 | 2.40E-15 |
| KAS | Scaffold_2 | 145750000 | 0.0528 | 13117 | 0.0893 | -0.0366 | -5.9350 | 2.94E-09 |
| KAS | Scaffold_2 | 145775000 | 0.0615 | 4311 | 0.0916 | -0.0301 | -5.0199 | 5.17E-07 |
| KAS | Scaffold_2 | 163825000 | 0.0565 | 4115 | 0.0165 | 0.0401 | 4.9132 | 8.96E-07 |
| KAS | Scaffold_3 | 92775000 | 0.0653 | 4824 | 0.0155 | 0.0498 | 7.0917 | 1.32E-12 |
| KAS | Scaffold_3 | 92800000 | 0.0686 | 3475 | 0.0157 | 0.0529 | 7.5743 | 3.61E-14 |
| KAS | Scaffold_3 | 93800000 | 0.0599 | 1280 | 0.0204 | 0.0395 | 5.4532 | 4.95E-08 |
| KAS | Scaffold_3 | 97950000 | 0.0641 | 3442 | 0.0253 | 0.0388 | 5.3495 | 8.82E-08 |
| KAS | Scaffold_3 | 97975000 | 0.0565 | 1697 | 0.0208 | 0.0356 | 4.8410 | 1.29E-06 |
| KAS | Scaffold_3 | 103350000 | 0.0613 | 1345 | 0.0189 | 0.0424 | 5.9187 | 3.24E-09 |
| KAS | Scaffold_3 | 106575000 | 0.0869 | 2251 | 0.0527 | 0.0342 | 4.6167 | 3.90E-06 |
| KAS | Scaffold_3 | 106600000 | 0.0869 | 2251 | 0.0527 | 0.0342 | 4.6167 | 3.90E-06 |
| KAS | Scaffold_3 | 134450000 | 0.0633 | 7070 | 0.0162 | 0.0470 | 6.6441 | 3.05E-11 |
| KAS | Scaffold_3 | 134475000 | 0.0506 | 6759 | 0.0109 | 0.0398 | 5.5007 | 3.78E-08 |
| KAS | Scaffold_3 | 140350000 | 0.1109 | 2796 | 0.0718 | 0.0390 | 5.3782 | 7.52E-08 |
| KAS | Scaffold_3 | 140425000 | 0.1049 | 1066 | 0.0708 | 0.0341 | 4.6030 | 4.17E-06 |
| KAS | Scaffold_3 | 140675000 | 0.0886 | 6509 | 0.0556 | 0.0331 | 4.4370 | 9.12E-06 |
| KAS | Scaffold_3 | 140850000 | 0.1041 | 2014 | 0.0691 | 0.0350 | 4.7465 | 2.07E-06 |
| KAS | Scaffold_3 | 141150000 | 0.1245 | 5923 | 0.0874 | 0.0370 | 5.0626 | 4.14E-07 |
| KAS | Scaffold_3 | 141200000 | 0.0951 | 2286 | 0.0614 | 0.0337 | 4.5441 | 5.52E-06 |
| KAS | Scaffold_3 | 161675000 | 0.0828 | 1163 | 0.0442 | 0.0386 | 5.3117 | 1.09E-07 |
| KAS | Scaffold_3 | 161700000 | 0.0544 | 2199 | 0.0182 | 0.0362 | 4.9268 | 8.36E-07 |
| KAS | Scaffold_4 | 54100000 | 0.0763 | 15414 | 0.0239 | 0.0524 | 5.6289 | 1.81E-08 |
| KAS | Scaffold_4 | 54125000 | 0.0918 | 8688 | 0.0255 | 0.0664 | 7.4198 | 1.17E-13 |
| KAS | Scaffold_4 | 60975000 | 0.0819 | 29231 | 0.0363 | 0.0456 | 4.7538 | 2.00E-06 |
| KAS | Scaffold_4 | 81475000 | 0.0952 | 8779 | 0.0501 | 0.0451 | 4.6852 | 2.80E-06 |
| KAS | Scaffold_5 | 29100000 | 0.0306 | 1237 | 0.0571 | -0.0265 | -4.8058 | 1.54E-06 |
| KAS | Scaffold_5 | 60125000 | 0.0852 | 1167 | 0.0491 | 0.0361 | 4.6291 | 3.67E-06 |
| KAS | Scaffold_5 | 63500000 | 0.0707 | 3090 | 0.0359 | 0.0348 | 4.4330 | 9.29E-06 |
| KAS | Scaffold_5 | 76025000 | 0.0610 | 1324 | 0.0251 | 0.0358 | 4.5835 | 4.57E-06 |
| KAS | Scaffold_5 | 77025000 | 0.0864 | 1500 | 0.0485 | 0.0378 | 4.8865 | 1.03E-06 |
| KAS | Scaffold_5 | 78850000 | 0.0527 | 1010 | 0.0146 | 0.0381 | 4.9230 | 8.52E-07 |
| KAS | Scaffold_5 | 86175000 | 0.0856 | 1060 | 0.0306 | 0.0550 | 7.4663 | 8.25E-14 |
| KAS | Scaffold_5 | 91975000 | 0.0756 | 1116 | 0.1037 | -0.0281 | -5.0587 | 4.22E-07 |
| KAS | Scaffold_5 | 102575000 | 0.0731 | 1905 | 0.0378 | 0.0353 | 4.4985 | 6.84E-06 |
| KAS | Scaffold_5 | 120525000 | 0.0280 | 1332 | 0.0527 | -0.0247 | -4.5376 | 5.69E-06 |
| KAS | Scaffold_5 | 120625000 | 0.0582 | 2850 | 0.0103 | 0.0479 | 6.4025 | 1.53E-10 |
| KAS | Scaffold_5 | 120650000 | 0.0573 | 2919 | 0.0104 | 0.0469 | 6.2512 | 4.07E-10 |
| KAS | Scaffold_5 | 142925000 | 0.0852 | 1177 | 0.0463 | 0.0389 | 5.0402 | 4.65E-07 |
| KAS | Scaffold_5 | 164775000 | 0.0519 | 2255 | 0.0971 | -0.0452 | -7.6221 | 2.50E-14 |
| KAS | Scaffold_5 | 164800000 | 0.0382 | 3571 | 0.0676 | -0.0294 | -5.2471 | 1.55E-07 |
| KAS | Scaffold_6 | 79450000 | 0.0197 | 3230 | 0.0461 | -0.0264 | -4.5246 | 6.05E-06 |
| KAS | Scaffold_6 | 94650000 | 0.0547 | 5228 | 0.0195 | 0.0351 | 4.5080 | 6.55E-06 |
| KAS | Scaffold_6 | 94750000 | 0.0565 | 11227 | 0.0194 | 0.0371 | 4.7939 | 1.64E-06 |
| KAS | Scaffold_6 | 96000000 | 0.0602 | 3183 | 0.0178 | 0.0424 | 5.5663 | 2.60E-08 |
| KAS | Scaffold_6 | 96025000 | 0.0796 | 3108 | 0.0121 | 0.0675 | 9.2504 | 2.24E-20 |
| KAS | Scaffold_6 | 96050000 | 0.0741 | 5831 | 0.0156 | 0.0585 | 7.9328 | 2.14E-15 |
| KAS | Scaffold_6 | 96075000 | 0.0573 | 4025 | 0.0206 | 0.0367 | 4.7361 | 2.18E-06 |
| KAS | Scaffold_6 | 96475000 | 0.0517 | 3954 | 0.0119 | 0.0398 | 5.1942 | 2.06E-07 |
| KAS | Scaffold_6 | 96500000 | 0.0510 | 3564 | 0.0097 | 0.0414 | 5.4237 | 5.84E-08 |
| KAS | Scaffold_6 | 158825000 | 0.0647 | 1525 | 0.0282 | 0.0365 | 4.6994 | 2.61E-06 |
| KAS | Scaffold_6 | 159275000 | 0.0689 | 1766 | 0.0264 | 0.0425 | 5.5864 | 2.32E-08 |
| KAS | Scaffold_6 | 159300000 | 0.0668 | 3741 | 0.0302 | 0.0366 | 4.7137 | 2.43E-06 |
| KAS | Scaffold_6 | 159325000 | 0.0734 | 6585 | 0.0317 | 0.0417 | 5.4729 | 4.43E-08 |
| KAS | Scaffold_6 | 159400000 | 0.0666 | 7605 | 0.0288 | 0.0378 | 4.8961 | 9.78E-07 |
| KAS | Scaffold_6 | 159425000 | 0.0658 | 6408 | 0.0309 | 0.0349 | 4.4738 | 7.68E-06 |
| KAS | Scaffold_6 | 159675000 | 0.0617 | 9149 | 0.0256 | 0.0361 | 4.6428 | 3.44E-06 |
| KAS | Scaffold_6 | 159700000 | 0.0624 | 2558 | 0.0196 | 0.0427 | 5.6217 | 1.89E-08 |
| KAS | Scaffold_6 | 159725000 | 0.0625 | 5279 | 0.0215 | 0.0410 | 5.3594 | 8.35E-08 |
| KAS | Scaffold_6 | 159750000 | 0.0677 | 4931 | 0.0264 | 0.0413 | 5.4034 | 6.54E-08 |
| KAS | Scaffold_6 | 159850000 | 0.0648 | 8036 | 0.0297 | 0.0351 | 4.5013 | 6.75E-06 |
| KAS | Scaffold_6 | 159925000 | 0.0607 | 6373 | 0.0211 | 0.0396 | 5.1680 | 2.37E-07 |
| KAS | Scaffold_6 | 159950000 | 0.0583 | 6149 | 0.0222 | 0.0360 | 4.6356 | 3.56E-06 |
| KAS | Scaffold_7 | 102725000 | 0.0555 | 1205 | 0.0796 | -0.0242 | -5.1319 | 2.87E-07 |
| KAS | Scaffold_7 | 106075000 | 0.0744 | 1526 | 0.0438 | 0.0307 | 4.4761 | 7.60E-06 |
| KAS | Scaffold_7 | 106100000 | 0.0780 | 15326 | 0.0459 | 0.0321 | 4.7282 | 2.27E-06 |
| KAS | Scaffold_7 | 106125000 | 0.0801 | 22731 | 0.0476 | 0.0325 | 4.7901 | 1.67E-06 |
| KAS | Scaffold_7 | 106150000 | 0.0826 | 14436 | 0.0488 | 0.0337 | 5.0064 | 5.55E-07 |
| KAS | Scaffold_7 | 106175000 | 0.0780 | 6594 | 0.0463 | 0.0317 | 4.6465 | 3.38E-06 |
| KAS | Scaffold_7 | 112950000 | 0.0593 | 1786 | 0.0264 | 0.0329 | 4.8599 | 1.17E-06 |
| KAS | Scaffold_7 | 113525000 | 0.0592 | 1002 | 0.0261 | 0.0331 | 4.9047 | 9.36E-07 |
| KAS | Scaffold_7 | 113700000 | 0.0852 | 2154 | 0.0513 | 0.0338 | 5.0283 | 4.95E-07 |
| KAS | Scaffold_7 | 113725000 | 0.0890 | 1130 | 0.0584 | 0.0306 | 4.4646 | 8.02E-06 |
| KAS | Scaffold_7 | 122950000 | 0.0468 | 1826 | 0.0716 | -0.0248 | -5.2453 | 1.56E-07 |
| KAS | Scaffold_8 | 75725000 | 0.0903 | 1083 | 0.0591 | 0.0313 | 5.3459 | 9.00E-08 |
| KAS | Scaffold_8 | 79425000 | 0.0887 | 1326 | 0.0624 | 0.0262 | 4.3677 | 1.26E-05 |
| KAS | Scaffold_8 | 82375000 | 0.0913 | 2410 | 0.0623 | 0.0290 | 4.9079 | 9.21E-07 |
| KAS | Scaffold_8 | 82400000 | 0.0865 | 2591 | 0.0600 | 0.0265 | 4.4097 | 1.04E-05 |
| KAS | Scaffold_8 | 83100000 | 0.0481 | 4191 | 0.0144 | 0.0337 | 5.8103 | 6.24E-09 |
| KAS | Scaffold_8 | 83125000 | 0.0455 | 4286 | 0.0132 | 0.0323 | 5.5431 | 2.97E-08 |
| KAS | Scaffold_8 | 100425000 | 0.0921 | 1214 | 0.0618 | 0.0304 | 5.1654 | 2.40E-07 |
| KAS | Scaffold_10 | 27375000 | 0.0507 | 4891 | 0.0208 | 0.0299 | 4.6765 | 2.92E-06 |
| KAS | Scaffold_10 | 49950000 | 0.0620 | 2650 | 0.0341 | 0.0279 | 4.3148 | 1.60E-05 |
| KAS | Scaffold_10 | 50100000 | 0.0518 | 10580 | 0.0205 | 0.0313 | 4.9347 | 8.03E-07 |
| KAS | Scaffold_10 | 50125000 | 0.0574 | 19491 | 0.0185 | 0.0389 | 6.2825 | 3.33E-10 |
| KAS | Scaffold_10 | 50150000 | 0.0654 | 23857 | 0.0256 | 0.0398 | 6.4378 | 1.21E-10 |
| KAS | Scaffold_10 | 50175000 | 0.0713 | 17184 | 0.0309 | 0.0404 | 6.5411 | 6.11E-11 |
| KAS | Scaffold_10 | 50200000 | 0.0687 | 4788 | 0.0262 | 0.0425 | 6.9143 | 4.70E-12 |
| KAS | Scaffold_10 | 85000000 | 0.0174 | 3509 | 0.0424 | -0.0250 | -5.0991 | 3.41E-07 |
| KAS | Scaffold_10 | 98425000 | 0.0299 | 6608 | 0.0517 | -0.0218 | -4.5253 | 6.03E-06 |
| KAS | Scaffold_10 | 101025000 | 0.0513 | 2801 | 0.0221 | 0.0292 | 4.5537 | 5.27E-06 |
| KAS | Scaffold_10 | 104325000 | 0.0525 | 1165 | 0.0243 | 0.0283 | 4.3845 | 1.16E-05 |
| KAS | Scaffold_11 | 46350000 | 0.0514 | 1538 | 0.0149 | 0.0365 | 4.8422 | 1.28E-06 |
| KAS | Scaffold_11 | 81625000 | 0.0618 | 3633 | 0.0268 | 0.0350 | 4.6026 | 4.17E-06 |
| KAS | Scaffold_12 | 15925000 | 0.0246 | 1814 | 0.0439 | -0.0193 | -6.1230 | 9.18E-10 |
| KAS | Scaffold_12 | 21125000 | 0.0191 | 4502 | 0.0344 | -0.0153 | -4.9165 | 8.81E-07 |
| KAS | Scaffold_12 | 24375000 | 0.0207 | 2710 | 0.0349 | -0.0142 | -4.5982 | 4.26E-06 |
| KAS | Scaffold_12 | 24400000 | 0.0144 | 1960 | 0.0300 | -0.0156 | -5.0155 | 5.29E-07 |
| KAS | Scaffold_12 | 35825000 | 0.0399 | 1528 | 0.0239 | 0.0161 | 4.4378 | 9.09E-06 |
| KAS | Scaffold_12 | 84250000 | 0.0180 | 5758 | 0.0351 | -0.0171 | -5.4669 | 4.58E-08 |
| KAS | Scaffold_12 | 95650000 | 0.0460 | 2594 | 0.0296 | 0.0163 | 4.5159 | 6.30E-06 |
| KAS | Scaffold_13 | 14700000 | 0.0109 | 1206 | 0.0425 | -0.0317 | -4.5964 | 4.30E-06 |
| KAS | Scaffold_13 | 17425000 | 0.0001 | 1302 | 0.0323 | -0.0322 | -4.6539 | 3.26E-06 |
| KAS | Scaffold_13 | 17675000 | 0.0018 | 2290 | 0.0318 | -0.0301 | -4.3933 | 1.12E-05 |
| KAS | Scaffold_13 | 17775000 | 0.0054 | 4360 | 0.0447 | -0.0393 | -5.5402 | 3.02E-08 |
| KAS | Scaffold_13 | 17800000 | 0.0018 | 2980 | 0.0525 | -0.0507 | -6.9737 | 3.09E-12 |
| KAS | Scaffold_13 | 17825000 | 0.0042 | 1446 | 0.0579 | -0.0537 | -7.3438 | 2.08E-13 |
| KAS | Scaffold_13 | 17850000 | 0.0077 | 1075 | 0.0619 | -0.0542 | -7.4044 | 1.32E-13 |
| KAS | Scaffold_13 | 18125000 | 0.0136 | 2528 | 0.0501 | -0.0364 | -5.1881 | 2.12E-07 |
| KAS | Scaffold_13 | 18175000 | 0.0216 | 3996 | 0.0514 | -0.0298 | -4.3568 | 1.32E-05 |
| KAS | Scaffold_13 | 78300000 | 0.0793 | 1973 | 0.0391 | 0.0403 | 4.3830 | 1.17E-05 |
| KAS | Scaffold_13 | 78325000 | 0.1015 | 2443 | 0.0444 | 0.0571 | 6.4790 | 9.23E-11 |
| KAS | Scaffold_13 | 84975000 | 0.0797 | 3787 | 0.0383 | 0.0414 | 4.5250 | 6.04E-06 |
| KAS | Scaffold_13 | 85850000 | 0.0950 | 1000 | 0.0545 | 0.0405 | 4.4101 | 1.03E-05 |
| KAS | Scaffold_13 | 86275000 | 0.0822 | 1411 | 0.0327 | 0.0495 | 5.5372 | 3.07E-08 |
| KAS | Scaffold_13 | 86350000 | 0.0870 | 3082 | 0.0417 | 0.0453 | 5.0141 | 5.33E-07 |
| KAS | Scaffold_13 | 86375000 | 0.0964 | 5343 | 0.0563 | 0.0401 | 4.3644 | 1.27E-05 |
| KAS | Scaffold_13 | 86400000 | 0.0993 | 10932 | 0.0565 | 0.0428 | 4.6935 | 2.69E-06 |
| KAS | Scaffold_13 | 86425000 | 0.0932 | 10467 | 0.0521 | 0.0412 | 4.4972 | 6.89E-06 |
| KAS | Scaffold_13 | 86825000 | 0.0912 | 2009 | 0.0473 | 0.0439 | 4.8413 | 1.29E-06 |
| KAS | Scaffold_14 | 9450000 | 0.0819 | 1911 | 0.0451 | 0.0368 | 4.5830 | 4.58E-06 |
| KAS | Scaffold_14 | 36750000 | 0.0109 | 1897 | 0.0430 | -0.0321 | -5.1537 | 2.55E-07 |
| KAS | Scaffold_14 | 36775000 | 0.0080 | 3132 | 0.0392 | -0.0313 | -5.0338 | 4.81E-07 |

**Supplementary Table 6.** Genomic windows showing significant differences in nucleotide diversity (Theta Pi) between the KIR and GER populations of *I. ricinus* across all 14 major scaffolds.

| **Pop** | **Chr** | **Pos** | **tP** | **nSites** | **Ger_tP** | **Diff_tP** | **Z_score** | **P_value** |
| --- | --- | --- | --- | --- | --- | --- | --- | --- |
| KIR | Scaffold_1 | 40275000 | 0.0575 | 14419 | 0.0197 | 0.0378 | 4.6482 | 3.35E-06 |
| KIR | Scaffold_1 | 100225000 | 0.0410 | 4409 | 0.0647 | -0.0238 | -4.5538 | 5.27E-06 |
| KIR | Scaffold_1 | 114125000 | 0.0568 | 10546 | 0.0096 | 0.0472 | 6.0551 | 1.40E-09 |
| KIR | Scaffold_1 | 114150000 | 0.0606 | 9862 | 0.0125 | 0.0481 | 6.1939 | 5.87E-10 |
| KIR | Scaffold_1 | 122275000 | 0.0603 | 3719 | 0.0234 | 0.0369 | 4.5154 | 6.32E-06 |
| KIR | Scaffold_1 | 122300000 | 0.0622 | 3515 | 0.0212 | 0.0410 | 5.1264 | 2.95E-07 |
| KIR | Scaffold_1 | 136150000 | 0.0719 | 5966 | 0.0336 | 0.0383 | 4.7257 | 2.29E-06 |
| KIR | Scaffold_1 | 136175000 | 0.0662 | 7850 | 0.0261 | 0.0402 | 5.0072 | 5.52E-07 |
| KIR | Scaffold_1 | 136200000 | 0.0581 | 4872 | 0.0207 | 0.0374 | 4.5856 | 4.53E-06 |
| KIR | Scaffold_1 | 139275000 | 0.0706 | 9799 | 0.0323 | 0.0383 | 4.7292 | 2.25E-06 |
| KIR | Scaffold_1 | 158000000 | 0.0702 | 6111 | 0.0217 | 0.0485 | 6.2513 | 4.07E-10 |
| KIR | Scaffold_1 | 158025000 | 0.0658 | 15094 | 0.0244 | 0.0414 | 5.1940 | 2.06E-07 |
| KIR | Scaffold_1 | 170075000 | 0.0520 | 2988 | 0.0118 | 0.0402 | 5.0059 | 5.56E-07 |
| KIR | Scaffold_1 | 170100000 | 0.0541 | 5416 | 0.0160 | 0.0381 | 4.6949 | 2.67E-06 |
| KIR | Scaffold_1 | 180050000 | 0.0660 | 6715 | 0.0254 | 0.0407 | 5.0824 | 3.73E-07 |
| KIR | Scaffold_1 | 180075000 | 0.0616 | 9909 | 0.0239 | 0.0377 | 4.6379 | 3.52E-06 |
| KIR | Scaffold_1 | 180125000 | 0.0652 | 11452 | 0.0246 | 0.0407 | 5.0781 | 3.81E-07 |
| KIR | Scaffold_1 | 180150000 | 0.0733 | 16464 | 0.0219 | 0.0514 | 6.6859 | 2.30E-11 |
| KIR | Scaffold_1 | 180175000 | 0.0704 | 13124 | 0.0245 | 0.0458 | 5.8495 | 4.93E-09 |
| KIR | Scaffold_1 | 180275000 | 0.0800 | 6038 | 0.0297 | 0.0502 | 6.5109 | 7.47E-11 |
| KIR | Scaffold_1 | 180300000 | 0.0607 | 2235 | 0.0214 | 0.0393 | 4.8804 | 1.06E-06 |
| KIR | Scaffold_2 | 36675000 | 0.0181 | 18496 | 0.0470 | -0.0289 | -4.5684 | 4.91E-06 |
| KIR | Scaffold_2 | 36700000 | 0.0141 | 16376 | 0.0460 | -0.0319 | -5.0003 | 5.72E-07 |
| KIR | Scaffold_2 | 44700000 | 0.0477 | 6105 | 0.0111 | 0.0366 | 4.6085 | 4.06E-06 |
| KIR | Scaffold_2 | 94850000 | 0.0713 | 1421 | 0.0200 | 0.0513 | 6.6646 | 2.65E-11 |
| KIR | Scaffold_2 | 129475000 | 0.0338 | 1304 | 0.0626 | -0.0288 | -4.5596 | 5.12E-06 |
| KIR | Scaffold_3 | 32475000 | 0.0816 | 8346 | 0.0537 | 0.0279 | 4.5533 | 5.28E-06 |
| KIR | Scaffold_3 | 35325000 | 0.0740 | 8454 | 0.0470 | 0.0270 | 4.3810 | 1.18E-05 |
| KIR | Scaffold_3 | 71550000 | 0.0338 | 1101 | 0.0558 | -0.0220 | -4.5285 | 5.94E-06 |
| KIR | Scaffold_3 | 92775000 | 0.0444 | 4824 | 0.0155 | 0.0289 | 4.7351 | 2.19E-06 |
| KIR | Scaffold_3 | 92800000 | 0.0478 | 3475 | 0.0157 | 0.0321 | 5.3167 | 1.06E-07 |
| KIR | Scaffold_3 | 140175000 | 0.1068 | 1415 | 0.0788 | 0.0281 | 4.5785 | 4.68E-06 |
| KIR | Scaffold_3 | 140350000 | 0.1006 | 2796 | 0.0718 | 0.0287 | 4.7026 | 2.57E-06 |
| KIR | Scaffold_3 | 140775000 | 0.1115 | 1822 | 0.0824 | 0.0291 | 4.7670 | 1.87E-06 |
| KIR | Scaffold_3 | 140800000 | 0.1135 | 1758 | 0.0836 | 0.0299 | 4.9201 | 8.65E-07 |
| KIR | Scaffold_3 | 140850000 | 0.1018 | 2014 | 0.0691 | 0.0327 | 5.4150 | 6.13E-08 |
| KIR | Scaffold_3 | 140875000 | 0.1034 | 2075 | 0.0745 | 0.0289 | 4.7286 | 2.26E-06 |
| KIR | Scaffold_4 | 37475000 | 0.0751 | 2251 | 0.0365 | 0.0386 | 4.6728 | 2.97E-06 |
| KIR | Scaffold_4 | 54125000 | 0.0666 | 8688 | 0.0255 | 0.0411 | 5.0387 | 4.69E-07 |
| KIR | Scaffold_4 | 80600000 | 0.0779 | 4990 | 0.0297 | 0.0482 | 6.0674 | 1.30E-09 |
| KIR | Scaffold_4 | 80625000 | 0.0839 | 6006 | 0.0380 | 0.0459 | 5.7384 | 9.56E-09 |
| KIR | Scaffold_4 | 111400000 | 0.0672 | 5475 | 0.0210 | 0.0462 | 5.7751 | 7.69E-09 |
| KIR | Scaffold_4 | 111425000 | 0.0693 | 11750 | 0.0261 | 0.0432 | 5.3429 | 9.15E-08 |
| KIR | Scaffold_4 | 165700000 | 0.0625 | 7084 | 0.0207 | 0.0419 | 5.1478 | 2.64E-07 |
| KIR | Scaffold_4 | 165725000 | 0.0653 | 9941 | 0.0247 | 0.0406 | 4.9705 | 6.68E-07 |
| KIR | Scaffold_4 | 165750000 | 0.0606 | 9428 | 0.0226 | 0.0380 | 4.5877 | 4.48E-06 |
| KIR | Scaffold_4 | 165775000 | 0.0589 | 9750 | 0.0207 | 0.0382 | 4.6193 | 3.85E-06 |
| KIR | Scaffold_4 | 165800000 | 0.0618 | 5266 | 0.0227 | 0.0391 | 4.7473 | 2.06E-06 |
| KIR | Scaffold_4 | 165925000 | 0.0607 | 3245 | 0.0227 | 0.0381 | 4.5972 | 4.28E-06 |
| KIR | Scaffold_5 | 53675000 | 0.0488 | 1060 | 0.0080 | 0.0408 | 5.4420 | 5.27E-08 |
| KIR | Scaffold_5 | 60125000 | 0.0842 | 1167 | 0.0491 | 0.0351 | 4.5918 | 4.39E-06 |
| KIR | Scaffold_5 | 86175000 | 0.0707 | 1060 | 0.0306 | 0.0401 | 5.3399 | 9.30E-08 |
| KIR | Scaffold_5 | 120625000 | 0.0582 | 2850 | 0.0103 | 0.0479 | 6.4952 | 8.29E-11 |
| KIR | Scaffold_5 | 120650000 | 0.0567 | 2919 | 0.0104 | 0.0463 | 6.2541 | 4.00E-10 |
| KIR | Scaffold_6 | 96025000 | 0.0732 | 3108 | 0.0121 | 0.0611 | 8.6903 | 3.62E-18 |
| KIR | Scaffold_6 | 96050000 | 0.0692 | 5831 | 0.0156 | 0.0536 | 7.5647 | 3.89E-14 |
| KIR | Scaffold_6 | 159325000 | 0.0656 | 6585 | 0.0317 | 0.0339 | 4.5890 | 4.45E-06 |
| KIR | Scaffold_6 | 159700000 | 0.0616 | 2558 | 0.0196 | 0.0419 | 5.8022 | 6.55E-09 |
| KIR | Scaffold_6 | 159725000 | 0.0571 | 5279 | 0.0215 | 0.0356 | 4.8398 | 1.30E-06 |
| KIR | Scaffold_6 | 159850000 | 0.0639 | 8036 | 0.0297 | 0.0342 | 4.6372 | 3.53E-06 |
| KIR | Scaffold_6 | 159925000 | 0.0581 | 6373 | 0.0211 | 0.0370 | 5.0568 | 4.26E-07 |
| KIR | Scaffold_6 | 159950000 | 0.0581 | 6149 | 0.0222 | 0.0358 | 4.8793 | 1.06E-06 |
| KIR | Scaffold_7 | 24075000 | 0.0631 | 1414 | 0.0336 | 0.0295 | 5.5367 | 3.08E-08 |
| KIR | Scaffold_7 | 24100000 | 0.0580 | 1457 | 0.0281 | 0.0300 | 5.6314 | 1.79E-08 |
| KIR | Scaffold_7 | 55425000 | 0.0465 | 1762 | 0.0720 | -0.0255 | -5.6980 | 1.21E-08 |
| KIR | Scaffold_7 | 55450000 | 0.0506 | 1440 | 0.0752 | -0.0246 | -5.5158 | 3.47E-08 |
| KIR | Scaffold_7 | 102775000 | 0.0409 | 5292 | 0.0670 | -0.0261 | -5.8343 | 5.40E-09 |
| KIR | Scaffold_7 | 102800000 | 0.0456 | 3598 | 0.0791 | -0.0335 | -7.3300 | 2.30E-13 |
| KIR | Scaffold_7 | 105275000 | 0.0619 | 1823 | 0.0372 | 0.0247 | 4.5551 | 5.24E-06 |
| KIR | Scaffold_7 | 105300000 | 0.0625 | 1269 | 0.0370 | 0.0255 | 4.7103 | 2.47E-06 |
| KIR | Scaffold_7 | 106075000 | 0.0744 | 1526 | 0.0438 | 0.0307 | 5.7700 | 7.93E-09 |
| KIR | Scaffold_7 | 106100000 | 0.0762 | 15326 | 0.0459 | 0.0303 | 5.6983 | 1.21E-08 |
| KIR | Scaffold_7 | 106125000 | 0.0766 | 22731 | 0.0476 | 0.0290 | 5.4287 | 5.68E-08 |
| KIR | Scaffold_7 | 106150000 | 0.0765 | 14436 | 0.0488 | 0.0276 | 5.1531 | 2.56E-07 |
| KIR | Scaffold_7 | 106175000 | 0.0740 | 6594 | 0.0463 | 0.0276 | 5.1549 | 2.54E-07 |
| KIR | Scaffold_7 | 113125000 | 0.0239 | 25318 | 0.0462 | -0.0223 | -5.0496 | 4.43E-07 |
| KIR | Scaffold_7 | 113525000 | 0.0540 | 1002 | 0.0261 | 0.0279 | 5.2093 | 1.90E-07 |
| KIR | Scaffold_8 | 2575000 | 0.0508 | 1030 | 0.0787 | -0.0280 | -5.5321 | 3.16E-08 |
| KIR | Scaffold_8 | 2600000 | 0.0517 | 1030 | 0.0796 | -0.0279 | -5.5252 | 3.29E-08 |
| KIR | Scaffold_8 | 33125000 | 0.0699 | 2473 | 0.0407 | 0.0293 | 5.3097 | 1.10E-07 |
| KIR | Scaffold_8 | 33150000 | 0.0699 | 2473 | 0.0407 | 0.0293 | 5.3097 | 1.10E-07 |
| KIR | Scaffold_8 | 44550000 | 0.0271 | 1141 | 0.0577 | -0.0306 | -6.0271 | 1.67E-09 |
| KIR | Scaffold_8 | 70550000 | 0.0481 | 2848 | 0.0164 | 0.0317 | 5.7730 | 7.79E-09 |
| KIR | Scaffold_8 | 70575000 | 0.0552 | 2676 | 0.0196 | 0.0356 | 6.5200 | 7.03E-11 |
| KIR | Scaffold_8 | 71250000 | 0.0619 | 9324 | 0.0366 | 0.0253 | 4.5521 | 5.31E-06 |
| KIR | Scaffold_8 | 71275000 | 0.0767 | 8731 | 0.0483 | 0.0284 | 5.1491 | 2.62E-07 |
| KIR | Scaffold_8 | 111825000 | 0.0425 | 3041 | 0.0653 | -0.0227 | -4.5398 | 5.63E-06 |
| KIR | Scaffold_9 | 38350000 | 0.0136 | 5605 | 0.0354 | -0.0218 | -4.4371 | 9.12E-06 |
| KIR | Scaffold_9 | 75425000 | 0.0676 | 2074 | 0.0344 | 0.0332 | 5.4585 | 4.80E-08 |
| KIR | Scaffold_10 | 50200000 | 0.0499 | 4788 | 0.0262 | 0.0237 | 4.5662 | 4.97E-06 |
| KIR | Scaffold_10 | 63950000 | 0.0651 | 2382 | 0.0373 | 0.0278 | 5.4273 | 5.72E-08 |
| KIR | Scaffold_10 | 63975000 | 0.0628 | 2833 | 0.0391 | 0.0238 | 4.5718 | 4.84E-06 |
| KIR | Scaffold_10 | 81275000 | 0.0529 | 2881 | 0.0283 | 0.0246 | 4.7437 | 2.10E-06 |
| KIR | Scaffold_10 | 81300000 | 0.0661 | 1867 | 0.0269 | 0.0392 | 7.8399 | 4.51E-15 |
| KIR | Scaffold_10 | 101025000 | 0.0480 | 2801 | 0.0221 | 0.0259 | 5.0188 | 5.20E-07 |
| KIR | Scaffold_11 | 56575000 | 0.1019 | 1270 | 0.0617 | 0.0402 | 5.0848 | 3.68E-07 |
| KIR | Scaffold_12 | 6125000 | 0.0344 | 1530 | 0.0494 | -0.0150 | -4.9239 | 8.48E-07 |
| KIR | Scaffold_12 | 15925000 | 0.0296 | 1814 | 0.0439 | -0.0143 | -4.6921 | 2.70E-06 |
| KIR | Scaffold_12 | 24150000 | 0.0657 | 1913 | 0.0487 | 0.0171 | 5.9358 | 2.92E-09 |
| KIR | Scaffold_12 | 26400000 | 0.0103 | 7329 | 0.0274 | -0.0172 | -5.6523 | 1.58E-08 |
| KIR | Scaffold_12 | 26425000 | 0.0141 | 5737 | 0.0383 | -0.0243 | -8.0545 | 7.98E-16 |
| KIR | Scaffold_12 | 26450000 | 0.0150 | 2814 | 0.0284 | -0.0134 | -4.3886 | 1.14E-05 |
| KIR | Scaffold_12 | 52275000 | 0.0887 | 2321 | 0.0734 | 0.0153 | 5.3317 | 9.73E-08 |
| KIR | Scaffold_12 | 102250000 | 0.0233 | 1298 | 0.0368 | -0.0136 | -4.4369 | 9.12E-06 |
| KIR | Scaffold_12 | 108875000 | 0.0612 | 1076 | 0.0479 | 0.0133 | 4.6538 | 3.26E-06 |
| KIR | Scaffold_13 | 78325000 | 0.0789 | 2443 | 0.0444 | 0.0345 | 4.4476 | 8.69E-06 |
| KIR | Scaffold_13 | 83300000 | 0.0762 | 2801 | 0.0349 | 0.0413 | 5.4064 | 6.43E-08 |
| KIR | Scaffold_13 | 84825000 | 0.0872 | 2814 | 0.0504 | 0.0368 | 4.7660 | 1.88E-06 |
| KIR | Scaffold_13 | 84975000 | 0.0762 | 3787 | 0.0383 | 0.0379 | 4.9229 | 8.53E-07 |
| KIR | Scaffold_13 | 85000000 | 0.0804 | 4688 | 0.0415 | 0.0389 | 5.0623 | 4.14E-07 |
| KIR | Scaffold_13 | 85025000 | 0.0889 | 1818 | 0.0424 | 0.0464 | 6.1258 | 9.02E-10 |
| KIR | Scaffold_13 | 85050000 | 0.0657 | 1705 | 0.0276 | 0.0381 | 4.9526 | 7.32E-07 |
| KIR | Scaffold_13 | 85575000 | 0.0895 | 3553 | 0.0530 | 0.0365 | 4.7284 | 2.26E-06 |
| KIR | Scaffold_13 | 85600000 | 0.0912 | 3008 | 0.0558 | 0.0354 | 4.5728 | 4.81E-06 |
| KIR | Scaffold_13 | 85850000 | 0.0934 | 1000 | 0.0545 | 0.0389 | 5.0594 | 4.21E-07 |
| KIR | Scaffold_13 | 86100000 | 0.0938 | 2527 | 0.0557 | 0.0381 | 4.9455 | 7.59E-07 |
| KIR | Scaffold_13 | 86125000 | 0.0762 | 1187 | 0.0423 | 0.0339 | 4.3562 | 1.32E-05 |
| KIR | Scaffold_13 | 86250000 | 0.0757 | 2006 | 0.0393 | 0.0365 | 4.7215 | 2.34E-06 |
| KIR | Scaffold_13 | 86275000 | 0.0804 | 1411 | 0.0327 | 0.0477 | 6.3005 | 2.97E-10 |
| KIR | Scaffold_13 | 86350000 | 0.0840 | 3082 | 0.0417 | 0.0423 | 5.5428 | 2.98E-08 |
| KIR | Scaffold_13 | 86375000 | 0.0929 | 5343 | 0.0563 | 0.0366 | 4.7343 | 2.20E-06 |
| KIR | Scaffold_13 | 86400000 | 0.0982 | 10932 | 0.0565 | 0.0417 | 5.4506 | 5.02E-08 |
| KIR | Scaffold_13 | 86425000 | 0.0967 | 10467 | 0.0521 | 0.0447 | 5.8742 | 4.25E-09 |
| KIR | Scaffold_13 | 86825000 | 0.0886 | 2009 | 0.0473 | 0.0413 | 5.3995 | 6.68E-08 |
| KIR | Scaffold_14 | 46225000 | 0.0424 | 1771 | 0.0100 | 0.0323 | 4.9503 | 7.41E-07 |
| KIR | Scaffold_14 | 48050000 | 0.0412 | 3566 | 0.0107 | 0.0306 | 4.6458 | 3.39E-06 |
| KIR | Scaffold_14 | 48075000 | 0.0377 | 4617 | 0.0068 | 0.0309 | 4.7087 | 2.49E-06 |
| KIR | Scaffold_14 | 55750000 | 0.0737 | 8687 | 0.0404 | 0.0333 | 5.1122 | 3.18E-07 |
| KIR | Scaffold_14 | 55775000 | 0.0641 | 11984 | 0.0331 | 0.0310 | 4.7187 | 2.37E-06 |
| KIR | Scaffold_14 | 55825000 | 0.0689 | 11663 | 0.0376 | 0.0313 | 4.7762 | 1.79E-06 |

**Supplementary Table 7.** Genomic windows showing significant differences in nucleotide diversity (Theta Pi) between the ORD and GER populations of *I. ricinus* across all 14 major scaffolds.

| **Pop** | **Chr** | **Pos** | **tP** | **nSites** | **Ger_tP** | **Diff_tP** | **Z_score** | **P_value** |
| --- | --- | --- | --- | --- | --- | --- | --- | --- |
| ORD | Scaffold_1 | 158000000 | 0.0781 | 6111 | 0.0217 | 0.0564 | 7.0051 | 2.47E-12 |
| ORD | Scaffold_1 | 158025000 | 0.0711 | 15094 | 0.0244 | 0.0467 | 5.6376 | 1.72E-08 |
| ORD | Scaffold_1 | 158050000 | 0.0664 | 9493 | 0.0273 | 0.0391 | 4.5584 | 5.15E-06 |
| ORD | Scaffold_1 | 170075000 | 0.0590 | 2988 | 0.0118 | 0.0471 | 5.6970 | 1.22E-08 |
| ORD | Scaffold_1 | 170100000 | 0.0625 | 5416 | 0.0160 | 0.0465 | 5.6064 | 2.07E-08 |
| ORD | Scaffold_1 | 170125000 | 0.0608 | 11448 | 0.0189 | 0.0419 | 4.9481 | 7.49E-07 |
| ORD | Scaffold_1 | 170150000 | 0.0622 | 14268 | 0.0196 | 0.0426 | 5.0604 | 4.18E-07 |
| ORD | Scaffold_1 | 170175000 | 0.0642 | 5811 | 0.0213 | 0.0428 | 5.0889 | 3.60E-07 |
| ORD | Scaffold_1 | 170950000 | 0.0694 | 1023 | 0.0292 | 0.0402 | 4.7106 | 2.47E-06 |
| ORD | Scaffold_1 | 180050000 | 0.0669 | 6715 | 0.0254 | 0.0416 | 4.9055 | 9.32E-07 |
| ORD | Scaffold_1 | 180075000 | 0.0656 | 9909 | 0.0239 | 0.0417 | 4.9247 | 8.45E-07 |
| ORD | Scaffold_1 | 180125000 | 0.0729 | 11452 | 0.0246 | 0.0483 | 5.8650 | 4.49E-09 |
| ORD | Scaffold_1 | 180150000 | 0.0766 | 16464 | 0.0219 | 0.0547 | 6.7692 | 1.29E-11 |
| ORD | Scaffold_1 | 180175000 | 0.0716 | 13124 | 0.0245 | 0.0471 | 5.6865 | 1.30E-08 |
| ORD | Scaffold_1 | 180275000 | 0.0851 | 6038 | 0.0297 | 0.0554 | 6.8615 | 6.82E-12 |
| ORD | Scaffold_1 | 180300000 | 0.0695 | 2235 | 0.0214 | 0.0481 | 5.8342 | 5.41E-09 |
| ORD | Scaffold_1 | 225950000 | 0.0647 | 1301 | 0.0249 | 0.0398 | 4.6548 | 3.24E-06 |
| ORD | Scaffold_1 | 225975000 | 0.0713 | 2482 | 0.0283 | 0.0430 | 5.1121 | 3.19E-07 |
| ORD | Scaffold_2 | 51750000 | 0.0821 | 2233 | 0.0374 | 0.0447 | 5.5148 | 3.49E-08 |
| ORD | Scaffold_2 | 51775000 | 0.0778 | 6628 | 0.0362 | 0.0416 | 5.0684 | 4.01E-07 |
| ORD | Scaffold_2 | 51800000 | 0.0753 | 4685 | 0.0369 | 0.0384 | 4.6060 | 4.11E-06 |
| ORD | Scaffold_2 | 94850000 | 0.0722 | 1421 | 0.0200 | 0.0522 | 6.6014 | 4.07E-11 |
| ORD | Scaffold_2 | 147350000 | 0.0692 | 1338 | 0.0968 | -0.0275 | -4.8717 | 1.11E-06 |
| ORD | Scaffold_2 | 147375000 | 0.0698 | 1587 | 0.0972 | -0.0275 | -4.8596 | 1.18E-06 |
| ORD | Scaffold_3 | 525000 | 0.0576 | 4094 | 0.0208 | 0.0368 | 5.5556 | 2.77E-08 |
| ORD | Scaffold_3 | 45925000 | 0.0311 | 1138 | 0.0585 | -0.0274 | -5.8002 | 6.62E-09 |
| ORD | Scaffold_3 | 134450000 | 0.0678 | 7070 | 0.0162 | 0.0516 | 8.1646 | 3.22E-16 |
| ORD | Scaffold_3 | 134475000 | 0.0586 | 6759 | 0.0109 | 0.0477 | 7.4762 | 7.65E-14 |
| ORD | Scaffold_3 | 136725000 | 0.1174 | 17192 | 0.0871 | 0.0304 | 4.4124 | 1.02E-05 |
| ORD | Scaffold_3 | 137175000 | 0.1177 | 5677 | 0.0821 | 0.0356 | 5.3414 | 9.22E-08 |
| ORD | Scaffold_3 | 137200000 | 0.1141 | 6202 | 0.0827 | 0.0315 | 4.6077 | 4.07E-06 |
| ORD | Scaffold_3 | 149225000 | 0.0509 | 1990 | 0.0138 | 0.0371 | 5.6064 | 2.07E-08 |
| ORD | Scaffold_4 | 37475000 | 0.0798 | 2251 | 0.0365 | 0.0433 | 4.9787 | 6.40E-07 |
| ORD | Scaffold_4 | 64350000 | 0.0567 | 3612 | 0.0139 | 0.0429 | 4.9208 | 8.62E-07 |
| ORD | Scaffold_4 | 68725000 | 0.0882 | 1651 | 0.0391 | 0.0491 | 5.8020 | 6.55E-09 |
| ORD | Scaffold_4 | 111400000 | 0.0640 | 5475 | 0.0210 | 0.0430 | 4.9331 | 8.09E-07 |
| ORD | Scaffold_4 | 163775000 | 0.0657 | 7356 | 0.0179 | 0.0478 | 5.6224 | 1.88E-08 |
| ORD | Scaffold_4 | 163800000 | 0.0676 | 9489 | 0.0240 | 0.0435 | 5.0139 | 5.33E-07 |
| ORD | Scaffold_4 | 164425000 | 0.0723 | 8335 | 0.0302 | 0.0420 | 4.8039 | 1.56E-06 |
| ORD | Scaffold_4 | 164450000 | 0.0823 | 8396 | 0.0332 | 0.0491 | 5.8043 | 6.46E-09 |
| ORD | Scaffold_4 | 164475000 | 0.0655 | 6511 | 0.0206 | 0.0449 | 5.2082 | 1.91E-07 |
| ORD | Scaffold_4 | 165700000 | 0.0668 | 7084 | 0.0207 | 0.0461 | 5.3766 | 7.59E-08 |
| ORD | Scaffold_4 | 165725000 | 0.0658 | 9941 | 0.0247 | 0.0410 | 4.6617 | 3.14E-06 |
| ORD | Scaffold_4 | 165750000 | 0.0664 | 9428 | 0.0226 | 0.0438 | 5.0446 | 4.55E-07 |
| ORD | Scaffold_4 | 165775000 | 0.0670 | 9750 | 0.0207 | 0.0462 | 5.3953 | 6.84E-08 |
| ORD | Scaffold_4 | 165800000 | 0.0675 | 5266 | 0.0227 | 0.0447 | 5.1836 | 2.18E-07 |
| ORD | Scaffold_5 | 49925000 | 0.0507 | 1171 | 0.0068 | 0.0439 | 5.6147 | 1.97E-08 |
| ORD | Scaffold_5 | 49950000 | 0.0446 | 1493 | 0.0080 | 0.0365 | 4.5072 | 6.57E-06 |
| ORD | Scaffold_5 | 78850000 | 0.0676 | 1010 | 0.0146 | 0.0529 | 6.9804 | 2.94E-12 |
| ORD | Scaffold_5 | 86175000 | 0.0897 | 1060 | 0.0306 | 0.0591 | 7.9021 | 2.74E-15 |
| ORD | Scaffold_5 | 86200000 | 0.0692 | 1190 | 0.0273 | 0.0419 | 5.3231 | 1.02E-07 |
| ORD | Scaffold_5 | 102225000 | 0.0777 | 1178 | 0.0339 | 0.0437 | 5.5950 | 2.21E-08 |
| ORD | Scaffold_5 | 106275000 | 0.0799 | 1594 | 0.0328 | 0.0471 | 6.1006 | 1.06E-09 |
| ORD | Scaffold_5 | 106300000 | 0.0799 | 1594 | 0.0328 | 0.0471 | 6.1006 | 1.06E-09 |
| ORD | Scaffold_5 | 120625000 | 0.0464 | 2850 | 0.0103 | 0.0361 | 4.4483 | 8.66E-06 |
| ORD | Scaffold_5 | 120650000 | 0.0467 | 2919 | 0.0104 | 0.0363 | 4.4721 | 7.75E-06 |
| ORD | Scaffold_5 | 138375000 | 0.0629 | 3357 | 0.0257 | 0.0372 | 4.6146 | 3.94E-06 |
| ORD | Scaffold_5 | 138400000 | 0.0750 | 2446 | 0.0259 | 0.0491 | 6.4056 | 1.50E-10 |
| ORD | Scaffold_5 | 164775000 | 0.0732 | 2255 | 0.0971 | -0.0239 | -4.5841 | 4.56E-06 |
| ORD | Scaffold_6 | 12725000 | 0.0700 | 1235 | 0.0226 | 0.0474 | 7.9154 | 2.46E-15 |
| ORD | Scaffold_6 | 47800000 | 0.0356 | 1337 | 0.0532 | -0.0176 | -4.4541 | 8.43E-06 |
| ORD | Scaffold_6 | 49575000 | 0.0296 | 1774 | 0.0480 | -0.0183 | -4.5917 | 4.40E-06 |
| ORD | Scaffold_6 | 49600000 | 0.0475 | 2866 | 0.0665 | -0.0191 | -4.7284 | 2.26E-06 |
| ORD | Scaffold_6 | 96025000 | 0.0671 | 3108 | 0.0121 | 0.0549 | 9.3552 | 8.34E-21 |
| ORD | Scaffold_6 | 96050000 | 0.0623 | 5831 | 0.0156 | 0.0467 | 7.7826 | 7.10E-15 |
| ORD | Scaffold_6 | 96075000 | 0.0524 | 4025 | 0.0206 | 0.0318 | 4.9524 | 7.33E-07 |
| ORD | Scaffold_6 | 96125000 | 0.0400 | 1491 | 0.0103 | 0.0297 | 4.5516 | 5.32E-06 |
| ORD | Scaffold_6 | 96475000 | 0.0465 | 3954 | 0.0119 | 0.0346 | 5.4908 | 4.00E-08 |
| ORD | Scaffold_6 | 96500000 | 0.0437 | 3564 | 0.0097 | 0.0341 | 5.3866 | 7.18E-08 |
| ORD | Scaffold_7 | 44350000 | 0.0416 | 1704 | 0.0601 | -0.0185 | -4.4531 | 8.46E-06 |
| ORD | Scaffold_7 | 50275000 | 0.1063 | 1254 | 0.0750 | 0.0313 | 4.6926 | 2.70E-06 |
| ORD | Scaffold_7 | 57425000 | 0.0360 | 1138 | 0.0568 | -0.0208 | -4.8832 | 1.04E-06 |
| ORD | Scaffold_7 | 106075000 | 0.0757 | 1526 | 0.0438 | 0.0320 | 4.8228 | 1.42E-06 |
| ORD | Scaffold_7 | 106150000 | 0.0845 | 14436 | 0.0488 | 0.0356 | 5.4931 | 3.95E-08 |
| ORD | Scaffold_7 | 106175000 | 0.0824 | 6594 | 0.0463 | 0.0361 | 5.5823 | 2.37E-08 |
| ORD | Scaffold_7 | 125675000 | 0.0449 | 1549 | 0.0072 | 0.0376 | 5.8639 | 4.52E-09 |
| ORD | Scaffold_8 | 21675000 | 0.0378 | 4307 | 0.0610 | -0.0233 | -4.4208 | 9.83E-06 |
| ORD | Scaffold_8 | 44550000 | 0.0304 | 1141 | 0.0577 | -0.0273 | -5.0750 | 3.87E-07 |
| ORD | Scaffold_8 | 62550000 | 0.0585 | 2529 | 0.0267 | 0.0318 | 4.5347 | 5.77E-06 |
| ORD | Scaffold_8 | 80575000 | 0.0497 | 1339 | 0.0789 | -0.0292 | -5.3943 | 6.88E-08 |
| ORD | Scaffold_8 | 83550000 | 0.0894 | 2377 | 0.0583 | 0.0312 | 4.4374 | 9.11E-06 |
| ORD | Scaffold_8 | 104650000 | 0.0692 | 1203 | 0.0330 | 0.0361 | 5.2427 | 1.58E-07 |
| ORD | Scaffold_9 | 10300000 | 0.0690 | 1096 | 0.0309 | 0.0381 | 5.3950 | 6.85E-08 |
| ORD | Scaffold_9 | 49075000 | 0.0506 | 1139 | 0.0174 | 0.0331 | 4.5774 | 4.71E-06 |
| ORD | Scaffold_9 | 55450000 | 0.0733 | 3957 | 0.0414 | 0.0319 | 4.3730 | 1.23E-05 |
| ORD | Scaffold_9 | 65750000 | 0.0656 | 3420 | 0.0340 | 0.0316 | 4.3335 | 1.47E-05 |
| ORD | Scaffold_10 | 27375000 | 0.0518 | 4891 | 0.0208 | 0.0310 | 4.9089 | 9.16E-07 |
| ORD | Scaffold_10 | 32425000 | 0.0605 | 1145 | 0.0318 | 0.0287 | 4.4685 | 7.88E-06 |
| ORD | Scaffold_10 | 50100000 | 0.0494 | 10580 | 0.0205 | 0.0290 | 4.5222 | 6.12E-06 |
| ORD | Scaffold_10 | 50125000 | 0.0510 | 19491 | 0.0185 | 0.0325 | 5.2006 | 1.99E-07 |
| ORD | Scaffold_10 | 50150000 | 0.0572 | 23857 | 0.0256 | 0.0316 | 5.0289 | 4.93E-07 |
| ORD | Scaffold_10 | 50175000 | 0.0626 | 17184 | 0.0309 | 0.0317 | 5.0489 | 4.44E-07 |
| ORD | Scaffold_10 | 50200000 | 0.0596 | 4788 | 0.0262 | 0.0334 | 5.3684 | 7.94E-08 |
| ORD | Scaffold_10 | 51325000 | 0.0107 | 1729 | 0.0290 | -0.0184 | -4.5085 | 6.53E-06 |
| ORD | Scaffold_10 | 111300000 | 0.0327 | 6360 | 0.0510 | -0.0182 | -4.4821 | 7.39E-06 |
| ORD | Scaffold_11 | 52475000 | 0.0793 | 2215 | 0.0417 | 0.0376 | 4.4542 | 8.42E-06 |
| ORD | Scaffold_11 | 52500000 | 0.0791 | 1486 | 0.0390 | 0.0401 | 4.8365 | 1.32E-06 |
| ORD | Scaffold_11 | 53425000 | 0.0995 | 1949 | 0.0552 | 0.0444 | 5.4636 | 4.67E-08 |
| ORD | Scaffold_11 | 53450000 | 0.1028 | 1859 | 0.0573 | 0.0455 | 5.6411 | 1.69E-08 |
| ORD | Scaffold_11 | 54450000 | 0.0729 | 3998 | 0.0336 | 0.0393 | 4.7153 | 2.41E-06 |
| ORD | Scaffold_11 | 54475000 | 0.0775 | 4584 | 0.0381 | 0.0394 | 4.7224 | 2.33E-06 |
| ORD | Scaffold_11 | 56575000 | 0.1031 | 1270 | 0.0617 | 0.0414 | 5.0180 | 5.22E-07 |
| ORD | Scaffold_11 | 56875000 | 0.1078 | 1044 | 0.0686 | 0.0392 | 4.7016 | 2.58E-06 |
| ORD | Scaffold_12 | 150000 | 0.0430 | 1794 | 0.0223 | 0.0207 | 5.2859 | 1.25E-07 |
| ORD | Scaffold_12 | 24125000 | 0.0533 | 3263 | 0.0352 | 0.0181 | 4.5146 | 6.34E-06 |
| ORD | Scaffold_12 | 24150000 | 0.0714 | 1913 | 0.0487 | 0.0228 | 5.8753 | 4.22E-09 |
| ORD | Scaffold_12 | 52275000 | 0.0954 | 2321 | 0.0734 | 0.0220 | 5.6597 | 1.52E-08 |
| ORD | Scaffold_12 | 52300000 | 0.0854 | 2798 | 0.0658 | 0.0196 | 4.9656 | 6.85E-07 |
| ORD | Scaffold_12 | 69275000 | 0.0476 | 2730 | 0.0294 | 0.0182 | 4.5547 | 5.24E-06 |
| ORD | Scaffold_12 | 84225000 | 0.0158 | 3808 | 0.0300 | -0.0142 | -4.9153 | 8.87E-07 |
| ORD | Scaffold_12 | 84250000 | 0.0152 | 5758 | 0.0351 | -0.0200 | -6.6069 | 3.92E-11 |
| ORD | Scaffold_13 | 72900000 | 0.1143 | 1974 | 0.0788 | 0.0355 | 4.6022 | 4.18E-06 |
| ORD | Scaffold_13 | 78325000 | 0.0785 | 2443 | 0.0444 | 0.0341 | 4.3917 | 1.12E-05 |
| ORD | Scaffold_13 | 80575000 | 0.0597 | 1270 | 0.0207 | 0.0390 | 5.1444 | 2.68E-07 |
| ORD | Scaffold_13 | 80600000 | 0.0522 | 2774 | 0.0162 | 0.0360 | 4.6901 | 2.73E-06 |
| ORD | Scaffold_13 | 80625000 | 0.0544 | 3140 | 0.0198 | 0.0346 | 4.4712 | 7.78E-06 |
| ORD | Scaffold_13 | 80650000 | 0.0697 | 1321 | 0.0269 | 0.0428 | 5.7459 | 9.14E-09 |
| ORD | Scaffold_13 | 100700000 | 0.0699 | 2792 | 0.0333 | 0.0366 | 4.7763 | 1.79E-06 |
| ORD | Scaffold_14 | 29025000 | 0.0646 | 1558 | 0.0246 | 0.0400 | 5.0074 | 5.52E-07 |
| ORD | Scaffold_14 | 58350000 | 0.0660 | 1942 | 0.0264 | 0.0395 | 4.9393 | 7.84E-07 |
| ORD | Scaffold_14 | 78575000 | 0.1107 | 1187 | 0.0700 | 0.0406 | 5.1130 | 3.17E-07 |
| ORD | Scaffold_14 | 79625000 | 0.0933 | 2905 | 0.0520 | 0.0413 | 5.2098 | 1.89E-07 |
